# Brain Activation by H_1_ Antihistamines Challenges Conventional View of Their Mechanism of Action in Motion Sickness: A Behavioral, c-Fos and Physiological Study in *Suncus murinus* (House Musk Shrew)

**DOI:** 10.3389/fphys.2017.00412

**Published:** 2017-06-14

**Authors:** Longlong Tu, Zengbing Lu, Karolina Dieser, Christina Schmitt, Sze Wa Chan, Man P. Ngan, Paul L. R. Andrews, Eugene Nalivaiko, John A. Rudd

**Affiliations:** ^1^School of Biomedical Sciences, Faculty of Medicine, The Chinese University of Hong KongHong Kong, China; ^2^Department of Informatics and Microsystem Technology, University of Applied Sciences KaiserslauternZweibrücken, Germany; ^3^School of Health Sciences, Caritas Institute of Higher EducationHong Kong, China; ^4^Division of Biomedical Sciences, St. George's University of LondonLondon, United Kingdom; ^5^School of Biomedical Sciences and Pharmacy, University of NewcastleCallaghan, NSW, Australia; ^6^Brain and Mind Institute, The Chinese University of Hong KongHong Kong, China

**Keywords:** gastric myoelectric activity, histamine H_1_ receptors, hypothermia, motion sickness, muscarinic receptors, respiration pattern, *Suncus murinus*

## Abstract

Motion sickness occurs under a variety of circumstances and is common in the general population. It is usually associated with changes in gastric motility, and hypothermia, which are argued to be surrogate markers for nausea; there are also reports that respiratory function is affected. As laboratory rodents are incapable of vomiting, *Suncus murinus* was used to model motion sickness and to investigate changes in gastric myoelectric activity (GMA) and temperature homeostasis using radiotelemetry, whilst also simultaneously investigating changes in respiratory function using whole body plethysmography. The anti-emetic potential of the highly selective histamine H_1_ receptor antagonists, mepyramine (brain penetrant), and cetirizine (non-brain penetrant), along with the muscarinic receptor antagonist, scopolamine, were investigated in the present study. On isolated ileal segments from *Suncus murinus*, both mepyramine and cetirizine non-competitively antagonized the contractile action of histamine with pK_*b*_ values of 7.5 and 8.4, respectively; scopolamine competitively antagonized the contractile action of acetylcholine with pA_2_ of 9.5. In responding animals, motion (1 Hz, 4 cm horizontal displacement, 10 min) increased the percentage of the power of bradygastria, and decreased the percentage power of normogastria whilst also causing hypothermia. Animals also exhibited an increase in respiratory rate and a reduction in tidal volume. Mepyramine (50 mg/kg, i.p.) and scopolamine (10 mg/kg, i.p.), but not cetirizine (10 mg/kg, i.p.), significantly antagonized motion-induced emesis but did not reverse the motion-induced disruptions of GMA, or hypothermia, or effects on respiration. Burst analysis of plethysmographic-derived waveforms showed mepyramine also had increased the inter-retch+vomit frequency, and emetic episode duration. Immunohistochemistry demonstrated that motion alone did not induce c-fos expression in the brain. Paradoxically, mepyramine increased c-fos in brain areas regulating emesis control, and caused hypothermia; it also appeared to cause sedation and reduced the dominant frequency of slow waves. In conclusion, motion-induced emesis was associated with a disruption of GMA, respiration, and hypothermia. Mepyramine was a more efficacious anti-emetic than cetirizine, suggesting an important role of centrally-located H_1_ receptors. The ability of mepyramine to elevate c-fos provides a new perspective on how H_1_ receptors are involved in mechanisms of emesis control.

## Introduction

Motion sickness, also known as kinetosis and travel sickness, is a common but complex syndrome which is characterized by a cluster of signs and symptoms including cold sweating, facial pallor, drowsiness, hypersalivation, “stomach awareness”, and nausea and vomiting (Golding and Gresty, 2015). Symptomology is very inter-individual variable (Sharma, 1997; Golding, 2006; Murdin et al., 2011) and there is no standardized method of assessment (Shupak and Gordon, 2006; Murdin et al., 2011). The most widely accepted mechanism of motion sickness is the “sensory-mismatch theory” which proposes motion-generated sensory conflict and neural mismatch between converging vestibular, visual and proprioceptive input patterns, that are different from learned and expected sensory patterns (Reason and Brand, 1975; Reason, 1978); for a discussion of other theories or modifications of the “sensory-mismatch theory” (see Oman, 2012; Oman and Cullen, 2014; Bertolini and Straumann, 2016). Irrespective of how sensory mismatch occurs, our understanding of how conflicted signal activate the pathways responsible for the induction of nausea and vomiting and accompanying physiological response, particularly in the stomach, is not well defined (Yates et al., 2014).

Two main classes of drug, anticholinergics (e.g., scopolamine) and antihistamines (e.g., promethazine) are the most common treatments for motion sickness (Schmäl, 2013; Golding and Gresty, 2015). However, these types of agents are variably efficacious in motion sickness and are associated with unwanted side effects including sedation, drowsiness, blurred vision, depression, and dry mouth/nose/throat (Spinks and Wasiak, 2011; Schmäl, 2013). Furthermore, the efficacy of all existing anti-motion sickness drugs is quite modest. The antihistamines used in humans to treat motion sickness are brain penetrant and are also weak muscarinic receptor antagonists (Simon and Simons, 2008; Schmäl, 2013). Compounds that do not penetrate the blood brain barrier have also been examined for their anti-motion sickness potential in humans. For example, the non-brain penetrant H_1_ receptor antagonist, terfenadine, possessing affinity for H_1_ receptor (IC_50_ = 6 nM) (Benavides et al., 1995), suppressed motion-induced nausea and autonomic dysfunction (Kohl et al., 1991). However, other non-brain penetrant compounds such as cetirizine and fexofenadine (the active metabolite of terfenadine) failed to prevent motion sickness, although their side effect profiles were not documented (Cheung et al., 2003). In these clinical studies, the motion sickness-rating scores were related to “nausea” and not “vomiting.” It remains unknown, therefore, whether highly selective, non-brain penetrant histamine H_1_ receptor antagonists are able to affect vomiting, as opposed to nausea or associated physiological changes, in the absence of undesirable side effects.

*Suncus murinus* (house musk shrew) is an insectivore used to study mechanisms of motion-induced emesis in which brain penetrant older-generation histamine H_1_receptor antagonists and the muscarinic receptor antagonist scopolamine have efficacy (Ueno et al., 1988). Supporting evidence for involvement of histamine in emesis comes from investigations showing an induction of emesis by histamine (Bhargava and Dixit, 1968) and the presence of histamine and acetylcholine receptors in the vestibular system (for reviews see, Matsuoka et al., 1983; Soto and Vega, 2010). In the present studies, therefore, we used *Suncus murinus* to elucidate the potential of the non-brain penetrant H_1_ receptor antagonist, cetirizine (Chen, 2008), to antagonize motion-induced emesis in comparison with the brain penetrant, highly selective H_1_ receptor antagonist, mepyramine (Fitzsimons et al., 2004); scopolamine was used as a positive control (Nakayama et al., 2005). These experiments were performed in animals implanted with radiotelemetry devices to permit recording of the gastric myoelectric activity (GMA) and body temperature, since alteration of gastric slow waves and hypothermia has been associated with motion-induced nausea in humans (Stern et al., 1987; Nalivaiko et al., 2015). We also recorded respiratory function, which is also disturbed during nausea and interrupted during emesis (Cowings et al., 1986; Himi et al., 2004; Gavgani et al., 2016; Horn et al., 2016). The collection of physiological data in *Suncus murinus* was also done in conjunction with an assessment of behavior to quantify side effects and to provide an insight into behaviors that collectively may be indicative of “nausea” (Horn et al., 2011, 2013). At the end of the experiments, brains were processed for c-fos immunohistochemistry to identify which central pathways were activated by motion stimulus. *Suncus murinus* is not a commonly used laboratory species, so we also assessed the potency of the antagonists at histamine H_1_ and muscarinic receptors using isolated ileal tissue segments to pharmacologically characterize the compounds to be used and to extrapolate doses for the *in vivo* anti-emetic part of the study. It was anticipated that the detailed studies of GMA, temperature, respiratory function and behavior coupled with c-fos would provide a comprehensive understanding of mechanisms activated during provocative motion. The use of selective H_1_ antihistamines and scopolamine would provide benchmarks for future studies on motion sickness using novel agents.

## Methods

### Animals

Adult male *Suncus murinus* (60–75 g, *n* = 178, 108 of them used *in vitro* and 70 *in vivo*) were obtained from The Chinese University of Hong Kong and housed in a temperature-controlled room (24 ± 1°C). Artificial lighting was provided between 06:00 and 18:00 h. The relative humidity was maintained at 50 ± 5%. Water and dry pelleted cat chow (Feline Diet 5003, PMI® Feeds, St. Louis, USA) were given *ad libitum* unless otherwise stated. All experiments were conducted under license from the Government of the Hong Kong SAR and the Animal Experimentation Ethics Committee, The Chinese University of Hong Kong.

### Organ bath studies

All animals were fasted overnight before being killed by cervical dislocation. The whole intestine was then removed and placed immediately in freshly prepared Krebs' solution (118 mM NaCl, 4.7 mM KCl, 1.2 mM KH_2_PO_4_, 1.2 mM MgSO_4_ 7H_2_O, 2.5 mM CaCl_2_ 2H_2_O, 25 mM NaHCO_3_, and 10 mM glucose) and gassed with 95 % O_2_ and 5% CO_2_ at room temperature. The ileum was identified (Uchino et al., 2002) and a 1 cm segment was removed and mounted longitudinally under 0.5 g tension in a 10 ml organ bath containing Krebs' solution and gassed with 95% O_2_ and 5% CO_2_ at 37°C. Only one isolated ileal section was used from each animal (Chan et al., 2007). After an equilibration period of 30 min, KCl (120 mM) was added to provide a reference contractile response followed by a washout. Histamine (100 nM–10 mM) was then added in a cumulative manner using a 3–5 min dosing schedule. At the end of concentration-response curve, KCl (120 mM) was added to check the viability of ileal segments. Twelve animals were used in this part. The mechanism of histamine to induce changes in smooth muscle tension was also investigated briefly using tissues from 24 animals (*n* = 6/treatment). Saline (0.9 w/v), tetrodotoxin (TTX, 1 μM), atropine (1 μM), or hexamethonium (HEX, 500 μM), was added to the organ bath 20 min prior to the addition of histamine (300 μM; based on its EC_50_ values as determined from the previous study). To estimate the effect of mepyramine and cetirizine on histamine-induced contraction (doses cumulatively, as before), tissues were equilibrated with mepyramine (0, 10, 30, and 100 nM) or cetirizine (0, 10, 30, and 100 nM) for 60 min, with regular washings every 20 min; Similarly, the effect of scopolamine (0, 1, 3, and 10 nM) on acetylcholine-induced contraction was also estimated. Seventy two animals were used for these studies (*n* = 6/treatment). Isometric contractions of ileal tissues were recorded using Grass transducers via a Mac Lab® system (ADInstruments Pty Ltd., New South Wales, Australia) connected to a Power Macintosh G3 computer. Analytical software (Chart, version 6.1, ADInstruments New South Wales, Australia) was used to record data. The volume of drug solutions added to the organ bath was less than 0.3% of the total volume.

### Implantation of radiotelemetry transmitters

Animals (*n* = 58) were fasted overnight and then injected with buprenorphine (0.05 mg/kg, s.c. Temgesic®), and anesthesia was induced by ketamine (20 mg/kg, i.m.; Alfasan, Holland) and xylazine (3 mg/kg, i.m.; Alfasan, Holland), and maintained with 3 % isoflurane (Halocarbon Products Corporation, USA) in a 3:1 ratio of O_2_ to N_2_O using an anesthetic machine (Narkomed 2C, Drager, USA). They were then placed on a heating pad (UCI#390 Analog moist heating pad, Rebirth Medical & Design, Inc., Taiwan) and the level of anesthesia was assessed and monitored throughout the surgery by the absence of the pedal withdrawal reflex. Following a midline abdominal incision, the distal stomach was exposed. Two biopotential wires of an ETA-F20 transmitter (Data Sciences, Inc., USA) were inserted into the serosal wall of antrum. The body of the transmitter was placed subcutaneously on the dorsal aspect of the animal. The abdominal cavity was closed using a continuous suture for the muscle layer and a discontinuous suture for the skin; the initial incision was sprayed with a permeable spray dressing (Opsite®, Smith and Nephew, UK). After surgery, all animals were administered marbofloxacin (Marbocyl®, 2 mg/kg, s.c.) once per day for 3 days, and buprenorphine (0.05 mg/kg, s.c.) was given again 12 h after the first dose. Animals were allowed 7 days to recover from the surgical procedures.

### Experimental protocols

*Suncus murinus* were initially pre-screened for motion sensitivity. Briefly, animals were fasted overnight before being put into clear Perspex whole body plethysmography chamber (diameter, 19.1 cm; height, 14 cm; volume, 4,014.83 cm^3^; Data Sciences, Inc., USA) with airflow set at 2.5 L/min provided by bias flow generator (Data Sciences, Inc., USA) for 30 min habituation followed by being presented with 10 g food. One hour after feeding, animals were subjected to provocative motion (1 Hz, 4 cm horizontal displacement, 10 min) triggered by a shaker (Heidolph Promax, UK) followed by a further 1 h recording. A range of animal behaviors (see below), including emesis, body temperature, gastric myoelectric activity (GMA), and respiratory activity were recorded.

Seven days later, animals exhibiting emesis in pre-screening were used to assess the anti-emetic potential of the antihistamines and scopolamine. These animals were randomized into different treatment groups (group of saline, mepyramine, cetirizine, and scopolamine) using a Latin square design. Drugs or vehicle (saline 2 ml/kg, i.p.) were administrated 1 h before provocative motion (1 Hz, 4 cm horizontal displacement, 10 min) followed by a further 1 h recording; telemetric, respiratory and behavioral data were acquired through the whole recording period. Animals were then deeply anesthetized with pentobarbitone (80 mg/kg, i.p.) (Dorminal®, Alfasan, Woerden, Holland) and intracardially perfused with pre-cooled (4°C) saline (40 ml) followed by 4% paraformaldehyde (PFA) in phosphate-buffered saline (PBS, 80 ml). Brains were removed and post-fixed in 4% PFA overnight at 4°C. After fixation, brains were transferred into 15% sucrose/4% PFA for dehydration overnight or until they sank, then to 30% sucrose/4% PFA until they sank again before being placed in aluminum foil containers filled with O.C.T. compound (Tissue-Tek, Sakura, USA). Samples were then stored at −80°C until sectioning for c-fos immunohistochemistry. Four sets of 12 animals, 3 in each set, were used as negative controls of vehicle, mepyramine, cetirizine, and scopolamine, separately, which were subjected to the same protocol as the corresponding group, but without motion.

### c-Fos immunohistochemistry

The methodology for c-fos immunohistochemistry followed that previously used in studies of pathways implicated in emesis in *Suncus murinus* (Chan et al., 2013, 2014). In brief, frozen tissues were sectioned at 40 μm in the coronal plane using a freezing microtome and incubated at room temperature for 1 h in 0.03% H_2_O_2_. The free-floating sections were blocked with 1.5% normal goat serum containing 0.3% Triton X-100 in PBS (Vectastain Elite ABC kit, Vector Laboratories, Burlingame, USA) for 1 h. After washing three times with PBS, sections were then incubated with rabbit anti-c-fos antibody (1: 10,000; Oncogene Research Products, Cambridge, USA) for 48 h at 4°C. The sections were subsequently washed and incubated with secondary goat-anti-rabbit antibody (1:200; Vector Laboratories) for 1 h, followed by Vectastain avidin–biotin complex reagent for 1 h (1:100; Vectastain Elite ABC kit, Vector Laboratories, Burlingame, USA). c-Fos expression was visualized using a commercially available peroxidase substrate (Vector® VIP kit, Vector Laboratories, Burlingame, USA). The number of c-fos immunoreactive cells was counted manually using a Zeiss Axioskop 2 plus microscope (Carl Zeiss Inc. Thornwood, USA) equipped with a Zeiss Axiocam 2 camera. To quantify expression in brainstem and hypothalamic nuclei, three representative sections were selected in accordance with the stereotaxic atlas constructed from our previous studies (Chan et al., 2013, 2014). Specifically, the anterior-posterior coordinates (measured from lambda) of the sections in which c-Fos were counted: +5.32, +5.44, and +5.66 for the ventral medial nucleus of hypothalamus (VMH), dorsal medial nucleus of hypothalamus (DMH), peduncular part of lateral hypothalamus (PLH), and arcuate nucleus (Arc); +5.92, +6.04, and +6.28 for paraventricular nucleus of hypothalamus (PVH); +0.50, +0.62, and 0.74 for medial vestibular nucleus (MVe); −0.26, −0.14, and −0.02 for the area postrema (AP) and nucleus tractus solitarius (NTS). The individual who performed all the counts was unaware of the treatment that the animals had received.

### Data acquisition and analysis

#### Behavior

Emesis was characterized as rhythmic abdominal contractions that were either associated with oral expulsion of solid or liquid material from the gastro-intestinal tract (i.e., vomiting), or not associated with passage of material (i.e., retching). An episode of retching and/or vomiting was considered separate when the animal changed its location inside the plethysmography chamber, or when the interval between retches and/or vomits exceeded 2 s (Rudd et al., 1999). Emetic events changed the pressure waveforms in the chamber and this was analyzed using a “burst analysis” technique (see below). Behaviors recorded were: *sniffing* (animal draw in a scent or air through nasal cavity inside the chamber; *face washing* (animal scratching its face with its forelimbs); *chin on the floor* (animal scratching its floor with its chin); *scratching* (animal use its hind limbs scratch its body); *licking* (tongue protrusion and movement—occurring during lying flat, resting, and periods where the animals actively moved around the chamber); *lying flat* (animal lying down with its stomach fully on floor, with the appearance of sedation); *resting* (animal conscious, but not moving). All behaviors were recorded as episodes, except resting and lying flat, which were recorded in min. JWatcher 1.0 software (Macquarie University, Sydney, Australia) was used to record behaviors.

#### Radiotelemetry

GMA data were initially analyzed using Spike2® (Version 8.1, Cambridge Electronic Design, UK) with methods previously developed by our laboratory (Percie du Sert et al., 2010). In brief, the gastric slow waves were recorded with a sampling frequency of 1,000 Hz, which were then subsequently filtered in several steps to a 0.03–0.5 Hz (2–30 cycles min^−1^) window, and down-sampled to 10.24 Hz to remove cardiac and respiratory signals, and low-frequency artifacts such as movement. Fast Fourier transforms (FFTs, Hann window, 2048) were computed on successive 10-min sections of data, and the following parameters were used to characterize the GMA: dominant frequency (DF) is defined as the frequency bin with the highest power in the 2–24 cpm range; dominant power (DP) is the highest power in the 2–24 cpm range; the repartition of power in bradygastric 2 to DF−2 cpm, normal DF−2 to DF+2 cpm and tachygastric (DF+2–24 cpm) ranges (i.e., bradygastria, normogastria, and tachygastria). All data collected by radiotelemetry including core body temperature were calculated by taking average of the data per 10 min.

We also used advanced analytical techniques to examine the structure of the slow waves. Thus, multifractal detrended fluctuation analysis (MFDFA) was used to obtain singularity spectra (α) (Kantelhardt et al., 2002); the multifractal spectrum identifies the deviation in fractal structure with time, compared with large and small fluctuations (Ihlen, 2012). Generally, the multifractal spectrum [plot *f(*α*)* vs. α] of signals with multifractal organization have a concave downward curvature. The width (Δα = α_*max*_ − α_*min*_) of singularity spectra has been used to characterize the spectra, which is a measure of complexity of the multifractal process. We used Spike2® (Version 8.1, Cambridge Electronic Design, UK) with custom scripts to perform the MFDFA.

#### Respiration

Whole body plethysmography has been previously used in *Suncus murinus* to semi-automate the recording of emetic events (Tashiro et al., 2007), but the methodology described here enabled the first detailed quantification of respiratory parameters using compensated whole body plethysmography (500-05RevA, Data Sciences, Inc., USA). The system consisted of two transparent chambers, each equipped with a Validyne pressure transducer (600–900 mmHg), a temperature sensor (0–100°C), and a humidity sensor (0–100 %). All channel signals from the two chambers were collected using ACQ7700 Carrier and UniversalXE Signal Conditioner collected to a Micro 1401 data acquisition unit (Cambridge Electronic Design, UK). Signals were thereafter acquired and analyzed using Spike2® (Version 8.1, Cambridge Electronic Design, UK) running on a PC desktop computer. It should be noted that animal body temperature is a prerequisite for calculating the respiratory tidal volume, and real time body temperature data from the telemetry transmitters was used in the calculation using offline processing in Microsoft Excel 2010 (v14.0, USA).

**Figure 1:**
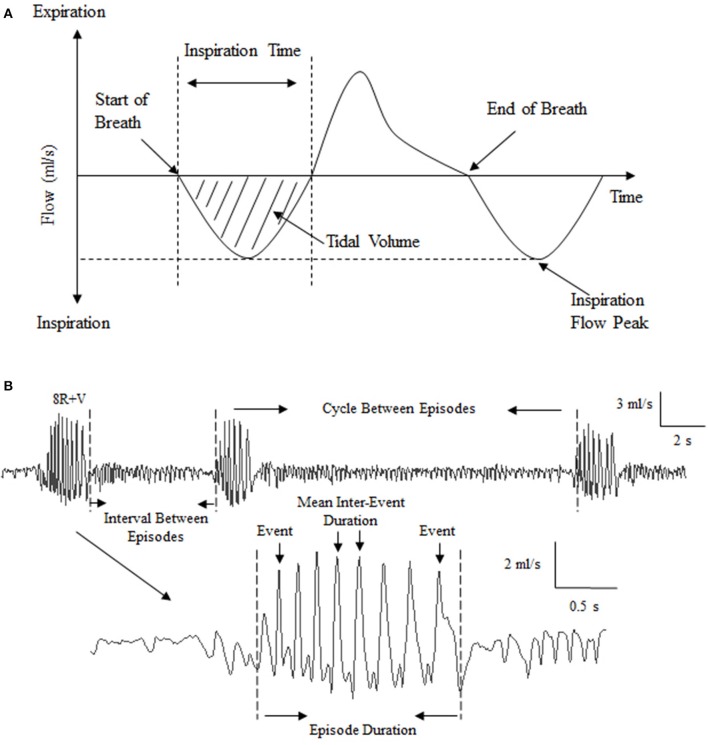
Illustration of the respiratory pattern of *Suncus murinus* and analysis of emetic data using burst analysis. **(A)** Illustration showing elements of the respiratory cycle (inspiration downwards). Mean respiratory rate, tidal volume, inspiration time and inspiration flow were used to characterize the respiratory pattern; **(B)** Illustration of the raw recording and analysis of emetic data using burst analysis. “Events” are large-amplitude single oscillatory cycles that coincided with visually observed contraction of abdominal muscles. Events per episode, mean inter-event duration, mean retch/vomit frequency, episode duration, interval between episodes (the duration from the end of last episode to the start of the next episode) and cycles between episodes (the duration from the onset of last episode to the start of the next episode) were defined to enable automated analysis of emetic episode data.

For characterizing the respiratory pattern, several parameters were used: respiratory rate, tidal volume, and inspiration time and flow, which was inclusive of regular respiratory activity and/or sniffing (See Figure 1A). Respiratory rate, which is the mean of the inter-breath interval, was computed from troughs in the respiratory signals. Respiratory data, excepting respiration rate, could not be collected during motion due to disruption of recordings caused by the shaking of the recording equipment. Therefore, only mean frequency was available to use for evaluation of respiratory function during motion. Moreover, respiratory signals were also discarded from the analysis during emetic episodes. All data were calculated by taking the average of the data per 2 min, before eventually averaging into 10 min segments for statistical analysis.

#### Analysis of emetic data using burst analysis

plethysmographic chamber pressure recordings: episodes of retching and/or vomiting interrupted and altered respiratory recordings (See Figure 1B). Usually, the inspiration flow for normal respiration is 0.2–1 ml/s. During a retch or vomit, the flow is approximately 3–5 ml/s, as evidenced by a cluster of sharp peaks (each peak representing a single retch or vomit) (Tashiro et al., 2007); these peaks were not necessarily related to lung volume, but more likely to represent a change of animal shape/volume during the physical process of retching or vomiting. Six parameters were defined to enable automated burst analysis using Spike2: events per episode, mean inter-event duration, mean retch/vomit frequency, episode duration, interval between episodes (the duration from the end of last episode to the start of the next episode) and cycle between episodes (the duration from the onset of last episode to the start of the next episode) (See Figure 1B).

### Statistical analyses

All statistical analyses were performed using Prism version 6.0 (GraphPad, California, USA). Difference between ileal tension, and all data obtained during pre-screening study including animal behavior and emesis, and all parameters of GMA and respiratory pattern were assessed using one-way ANOVA followed by a Bonferroni's multiple comparison tests. The method to calculate pA_2_ and pK_*b*_ values was described previously (Schild, 1947; Gaddum et al., 1955). The differences between animal behaviors, and all parameters of GMA, and respiration patterns among vehicle, mepyramine, cetirizine, and scopolamine groups were assessed using repeated two-way ANOVA (factors: time and treatment), followed by Bonferroni test. Pearson's correlation was used to assess correlations among episodes, DF, DP, body temperature, respiration rate, body weight, bradygastria, normogastria, tachygastria, and tidal volume values, which were obtained in the pre-screening study. During our analysis, baseline refers to a 10 min period immediately before provocation motion; recovery indicates 10 min immediately after provocation motion. All data are expressed as the mean ± s.e.m. Differences were considered significant when *p* < 0.05.

### Drug formulation

Histamine dihydrochloride, tetrodotoxin (TTX), atropine methylnitrate, hexamethonium bromide (HEX), and scopolamine hydrochloride were from Sigma-Aldrich, St. Louis, USA. Mepyramine maleate, cetirizine dihydrochloride were from Tocris, Bristol, UK. All drugs were dissolved in saline (0.9%, w/v).

## Results

### The effect of mepyramine and cetirizine on histamine-induced contractions of the isolated ileum

**Figure 2:**
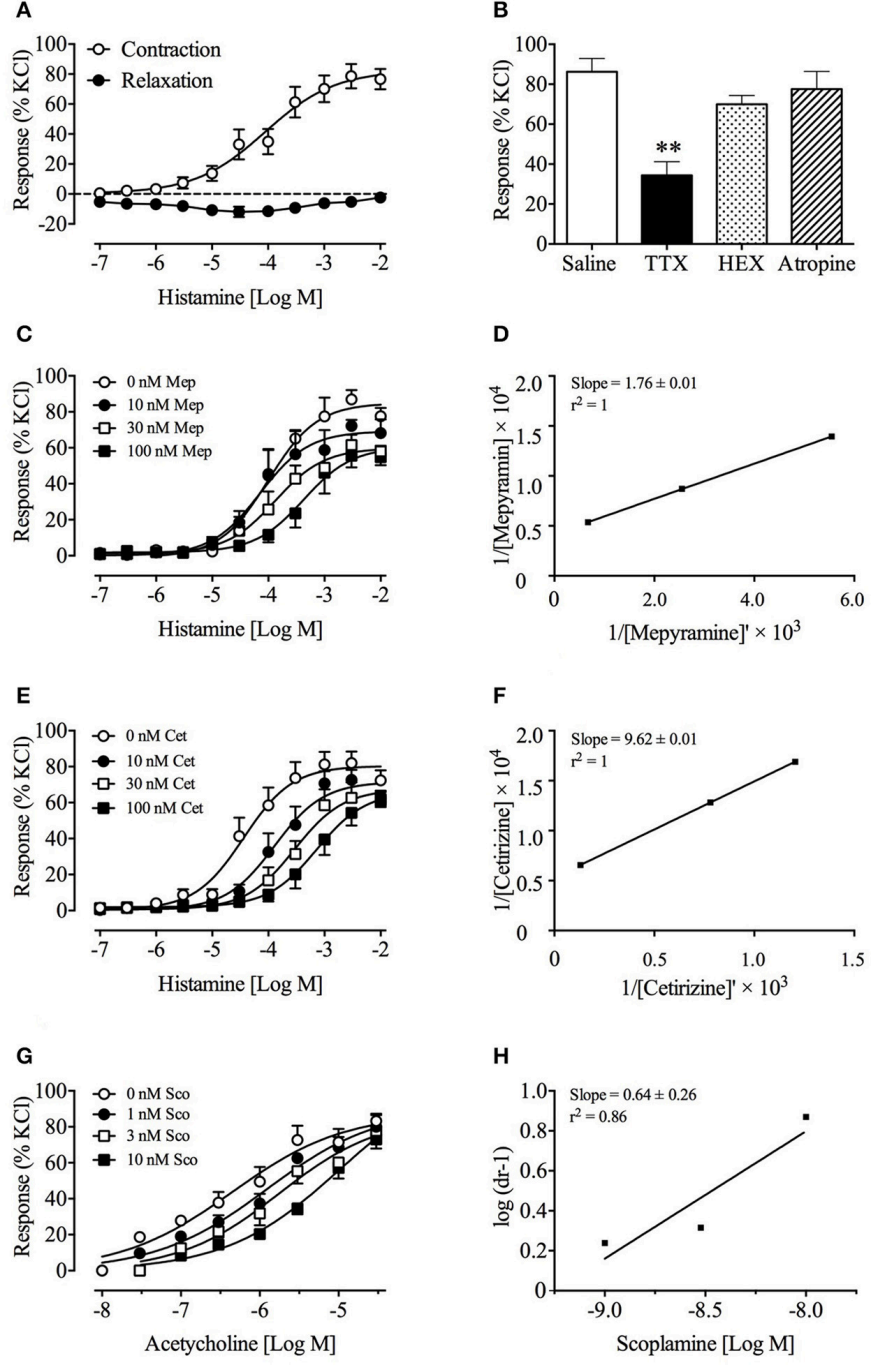
Effect of mepyramine and cetirizine on histamine-induced, and scopolamine on acetylcholine-induced contractions of *Suncus murinus* isolated ileal sections. **(A)** Concentration-response curve of histamine against ileal sections; **(B)** Effect of TTX, HEX, and atropine on histamine-induced contraction of isolated ileum; **(C,E)** Effect of mepyramine and cetirizine on histamine-induced contraction of isolated ileum; **(D,F)** Double reciprocal plot for histamine in the presence of 30 nM mepyramine and cetirizine, respectively; **(G)** Effect of scopolamine on acetylcholine-induced contraction of isolated ileum; **(H)** Schild analysis of scopolamine on acetylcholine-induced contractions. Data represents mean ± s.e.m. of 6–12 determinations. Significant differences compared to the control group are indicated as ^**^p < 0.01 (One-way ANOVA followed by Bonferroni test).

Histamine caused a small transient relaxation between 100 nM and 1 μM. At higher concentrations of 3 μM–10 mM, the response was biphasic, manifested as an initial transient relaxation followed by a sustained contraction. The contractile action had a pEC_50_ of 4.1 ± 0.2 with an E_*max*_ of 82.4 ± 8.3% (120 mM KCl response); the transient relaxation effect ranged from −2.4 ± 1.0 % to −11.5 ± 2.9 % (120 mM KCl response) (Figure 2A). The contractile action was significantly reduced by 52% by tetrodotoxin (1 μM) (*p* < 0.01), but not by atropine (1 μM) or hexamethonium (500 μM) (Figure 2B). Mepyramine (10–100 nM) and cetirizine (10–100 nM) caused a progressive rightward shift of the concentration-response curves to histamine, with depression of maxima (Figures 2C,E). Acetylcholine induced a contraction of the ileum, with a pEC_50_ of 6.3 ± 0.2 and an E_*max*_ of 87.5 ± 8.9 % (120 mM KCl response). Scopolamine (1–10 nM) caused a progressive rightward shift of the concentration-response curves to acetylcholine, without depressing the maxima (Figure 2G). Double reciprocal analysis of mepyramine and cetirizine, and Schild analysis of scopolamine plots yielded pK_*b*_ values of 7.5 and 8.4, and pA_2_ value of 9.5, respectively (Figures 2D,F,H). Mepyramine and cetirizine failed to affect the minor relaxant effect of histamine during these experiments.

### Behavior observations

*Suncus murinus* were initially pre-screened for motion sensitivity, of which 87.9% had emesis: in the responding animals the latency was 4.7 ± 0.4 min and there were 8.4 ± 0.6 episodes occurring with 4.3 ± 0.5 vomits and 35.3 ± 2.7 retches (*n* = 51). Motion induced a reduction in the number of episodes of sniffing (Table 1, *p* < 0.001) and increased the number of episodes of scratching (Table 1, *p* < 0.001), without affecting other behaviors in the 1 h period following motion, in comparison with the preceding 1 h basal behavior before motion. Animals became motionless soon after the onset of motion until the end of motion.

**Table 1:** Effect of provocative motion on spontaneous behaviors in *Suncus murinus*.

	**Spontaneous behaviors before motion (60 min)**	**Spontaneous behaviors after motion (60 min)**
Face washing	2.9 ± 0.5	2.0 ± 0.5
Chin on the floor	3.9 ± 1.1	3.7 ± 1.0
Scratching	17.7 ± 3.7	30.4 ± 4.8^***^
Sniffing	25.4 ± 3.1	12.0 ± 1.7^***^
Licking	3.2 ± 1.0	4.5 ± 1.7
Resting (min)	39.5 ± 3.0	37.8 ± 2.6

Resting is calculated in mins, other behaviors are calculated in episodes. Data represents the mean ± s.e.m. of 24 animals. Significant differences compared to baseline are shown as

*** *p < 0.001 (paired t-test)*.

### Effect of provocative motion on GMA, body temperature, and respiratory pattern of *Suncus murinus*

During pre-screening, the baseline DF was 14.7 ± 0.2 cycle/min (cpm) with a DP of 2.6 ± 0.4 ^*^ 10^−4^ mv^2^. 26.3 ± 1.9% of power was in the bradygastric range, 43.6 ± 2.6% of power was in the normogastric range, and 18.2 ± 1.2% of power was in the tachygastric range (*n* = 58). In the responding animals (*n* = 51), motion caused a 6.2% increase in the % power of the bradygastric range (Table 2, *p* < 0.01), whilst causing a 12.1% decrease the % power of the normogastric range (Table 2, *p* < 0.001). A slight fall in body temperature was also observed during motion (−0.3 ± 0.04°C) and recovery (−0.6 ± 0.1°C) (Table 2, *p* < 0.001). Animals exhibited a basal respiratory rate of 243 ± 24 breaths per minute (bpm), a tidal volume of 0.47 ± 0.02 ml, an inspiration time of 0.12 ± 0.01 s, and an inspiration flow of 0.58 ± 0.03 ml/s. There was a ~132 bpm increase (54% increase) of respiration rate (Table 2, *p* < 0.001) during motion. Compared to baseline, a ~129 bpm increase of respiration rate and a ~0.13 ml reduction in tidal volume (Table 2, *p* < 0.001), concomitant with a 0.04 s reduction in inspiration time (Table 2, *p* < 0.001) and a ~0.09 ml/s increase of inspiratory flow (Table 2, *p* < 0.001) were observed during recovery period.

**Table 2:** Effect of provocative motion on gastric myoelectric activity, body temperature, and respiratory pattern.

	**Baseline**	**Motion**	**Recovery**
DF (cpm)	14.67 ± 0.20	14.42 ± 0.28	15.06 ± 0.27
DP (^*^10^−4^ mv^2^)	2.59 ± 0.40	2.37 ± 0.33	2.96 ± 0.43
Bradygastria (%)	26.26 ± 1.88	32.45 ± 2.28^**^	28.28 ± 1.98
Normogastria (%)	43.55 ± 2.59	31.44 ± 2.17^***^	36.76 ± 2.67
Tachygastria (%)	18.21 ± 1.22	20.07 ± 1.32	21.63 ± 2.06
Body Temperature (°C)	34.70 ± 0.13	34.41 ± 0.13^***^	34.16 ± 0.16^***^
Respiration Rate (bpm)	243.30 ± 23.64	375.30 ± 16.10^***^	372.20 ± 20.52^***^
Tidal Volume (ml)	0.47 ± 0.02	–	0.34 ± 0.01^***^
Inspiration Time (s)	0.12 ± 0.01	–	0.08 ± 001^***^
Inspiration Flow (ml/s)	0.58 ± 0.03	–	0.67 ± 0.03^**^

Data represents the mean ± s.e.m. of 44–52 animals. Significant differences compared to baseline are shown as

** p < 0.01,

*** *p < 0.01 (One-way ANOVA followed by Bonferroni tests). Baseline refers to a 10 min period immediately before provocation motion; recovery indicates 10 min immediately after provocation motion*.

### Effect of mepyramine, cetirizine, and scopolamine on animal behavior and motion-induced emesis

Consistent with the pre-screening study, a range of animal behaviors were quantified following drug/vehicle administration. Sedation, represented by lying flat, was only observed following mepyramine (Table 3, *p* < 0.001), and the animals also spent less time resting (Table 3, *p* < 0.001). The mepyramine-treated animals also had fewer episodes of scratching compared to the vehicle group (Table 3, *p* < 0.001). Cetirizine and scopolamine did not affect the behavior of the animals (Table 3, *p* > 0.05).

**Table 3:** Effect of mepyramine (50 mg/kg), cetirizine (10 mg/kg), and scopolamine (10 mg/kg) on spontaneous behaviors in *Suncus murinus*.

	**Basal spontaneous behavior before motion (60 min)**				**Basal spontaneous behavior after motion (60 min)**			
	**Vehicle**	**Mepyramine**	**Cetirizine**	**Scopolamine**	**Vehicle**	**Mepyramine**	**Cetirizine**	**Scopolamine**
Face washing	5.8 ± 3.4	0.2 ± 0.4	2.8 ± 1.3	1.2 ± 0.6	1.7 ± 2.7	0.2 ± 0.4	1.3 ± 1.8	1.5 ± 0.8
Chin on the floor	13.5 ± 12.1	17.8 ± 15.8	5.0 ± 5.4	9.0 ± 4.4	1.3 ± 1.5	5.2 ± 6.2	1.2 ± 2.9	0.7 ± 0.3
Scratching	22.1 ± 9.0	1.3 ± 2.1^***^	9.3 ± 9.1	16.5 ± 5.5	15.7 ± 12.9	25.7 ± 17.4	8.8 ± 8.2	17.0 ± 8.0
Sniffing	33.3 ± 11.5	22.7 ± 7.8	28.5 ± 15.4	26.8 ± 9.0	9.3 ± 7.4	9.0 ± 8.9	7.0 ± 6.1	10.0 ± 4.4
Licking	4.5 ± 6.4	0.3 ± 0.5	1.7 ± 3.6	5.7 ± 1.9	2.8 ± 1.8	3.3 ± 7.2	2.3 ± 3.9	3.0 ± 1.5
Lying flat (min)	0	26.7 ± 12.7^***^	0	0	0	2.9 ± 6.7	0	0
Resting (min)	35.2 ± 9.1	7.7 ± 8.1^***^	42.6 ± 3.6	37.3 ± 4.5	50.2 ± 4.6	41.2 ± 12.6	39.2 ± 1.2	45.0 ± 3.3

Resting and lying flat are calculated in mins, other behaviors are calculated in episodes. Data represents the mean ± s.e.m. of 6 animals. Significant differences compared to vehicle group are shown as

*** *p < 0.001 (One-way ANOVA followed by Bonferroni tests)*.

**Figure 3:**
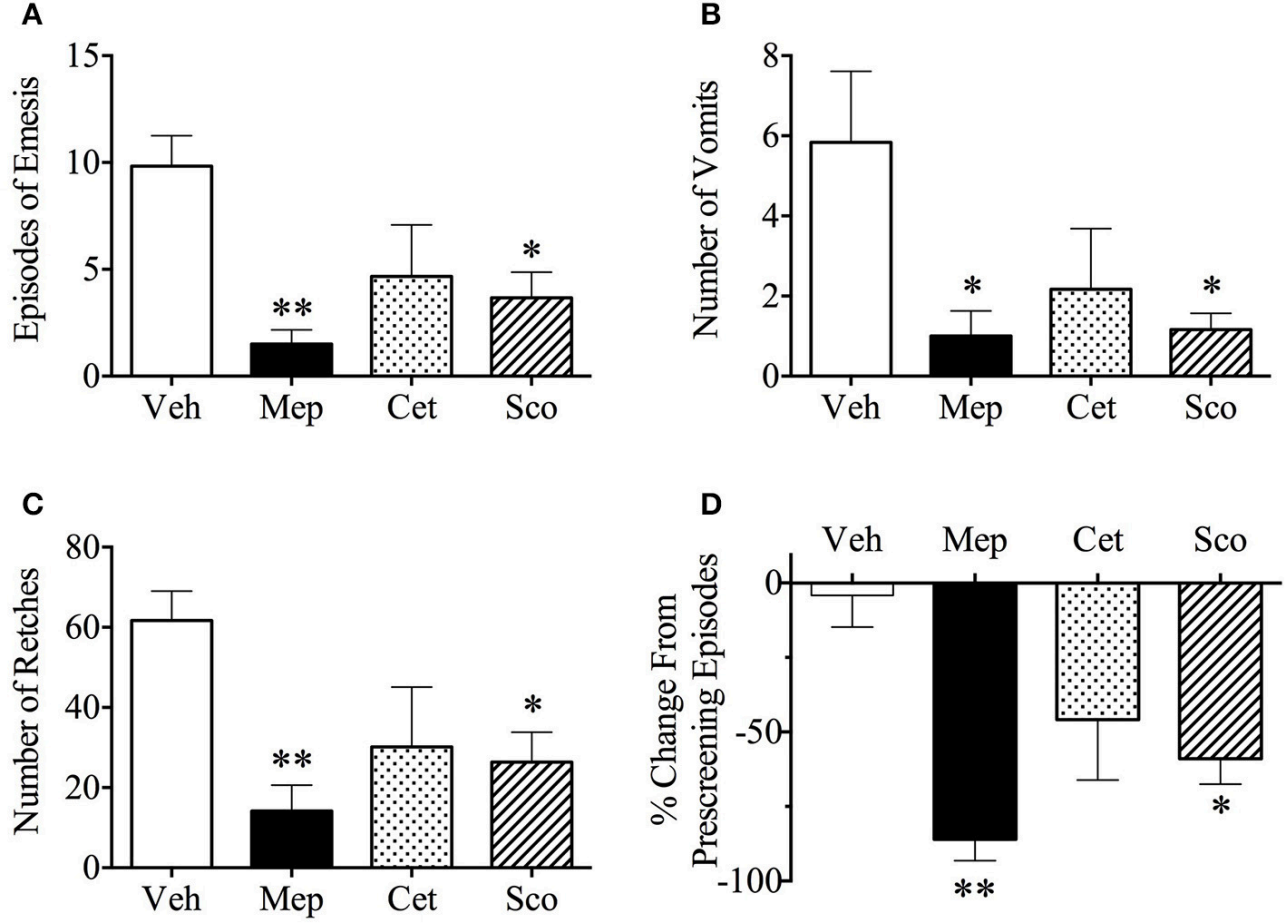
Effect of mepyramine (50 mg/kg), cetirizine (10 mg/kg), and scopolamine (10 mg/kg) on motion-induced emesis in *Suncus murinus*. **(A)** Number of episodes of emesis; **(B)** Number of vomits; **(C)** Number of retches; **(D)** % change from pre-screening episodes. Drug or vehicle was administered intraperitoneally as a 60 min pretreatment. Results represent the mean ± s.e.m. of 6 animals. Significant differences compared to vehicle group are shown as *p < 0.05, **p < 0.01 (One-way ANOVA followed by Bonferroni tests). Veh, vehicle; Mep, mepyramine; Cet, cetirizine; Sco, scopolamine.

Vehicle-treated animals exhibited 9.8 ± 1.4 episodes during provocative motion, which consisted of 5.8 ± 1.8 vomits and 61.7 ± 7.4 retches (Figures 3A–C, *n* = 6). Both mepyramine and scopolamine, but not cetirizine, significantly antagonized motion-induced emesis in terms of number of episodes, vomits, and retches (Figures 3A–C). When compared with the number of episodes of vomiting and/or retching during pre-screening, mepyramine and scopolamine caused a significant reduction by 86 ± 7.0% (*p* < 0.01) and 59 ± 8.5% (*p* < 0.05) as shown in Figure 3D. None of the treatments affected the latency to the first episode of retching and/or vomiting (vehicle, 3.7 min; mepyramine, 6.1 min; cetirizine, 4.7 min; and scopolamine, 5.6 min; median values).

### Burst analysis of emetic data

**Figure 4:**
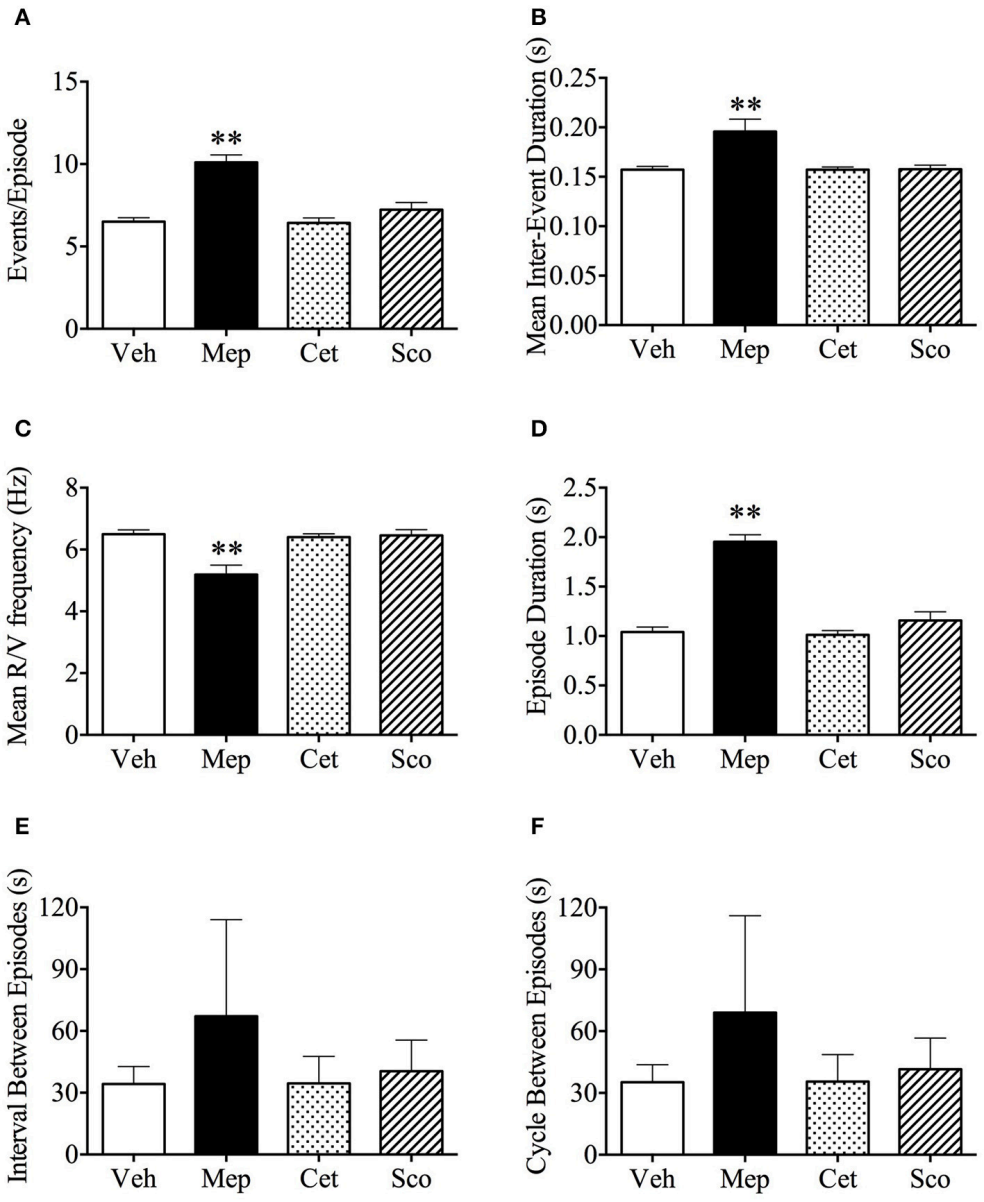
Analysis of emetic data using burst analysis. **(A)** Events per episode/burst; **(B)** Mean inter-event duration; **(C)** Mean retch/vomit frequency; **(D)** Episodes duration; **(E)** Interval between episodes; **(F)** Cycle between episodes. Results represent the mean ± s.e.m. of all animals which vomited (*n* = 3–6). Significant differences compared to vehicle group are shown as **p < 0.01 (One-way ANOVA followed by Bonferroni tests). Veh, vehicle (saline, 2 ml/kg); Mep, mepyramine (50 mg/kg); Cet, cetirizine (10 mg/kg); Sco, scopolamine (10 mg/kg).

In our experiments, a “retch/vomit” refers to a single high-amplitude wave of pressure change during an emetic episode (Figure 1B). Vehicle, cetirizine, and scopolamine-treated animals had 6.5 ± 0.2, 6.4 ± 0.3, and 7.2 ± 1.4 retches+vomits per emetic episode, respectively (Figure 4A, *n* = 6, *p* > 0.05). In comparison, the animals treated with mepyramine had 10.1 ± 0.4 retches+vomits per emetic episode, which was significantly higher than the other three groups (Figure 4A, *p* < 0.01). Similarly, the mean retches+vomits frequency, and emetic episode duration, all increased significantly (*p* < 0.01) in the mepyramine-treated group (Figures 4B–D). There was no difference between data for any of the treatment groups with respect to the intervals between episodes, or the cycles between episodes (Figures 4E,F, *p* > 0.05).

### Effect of mepyramine, cetirizine, and scopolamine on gastric myoelectric activity and body temperature

**Figure 5:**
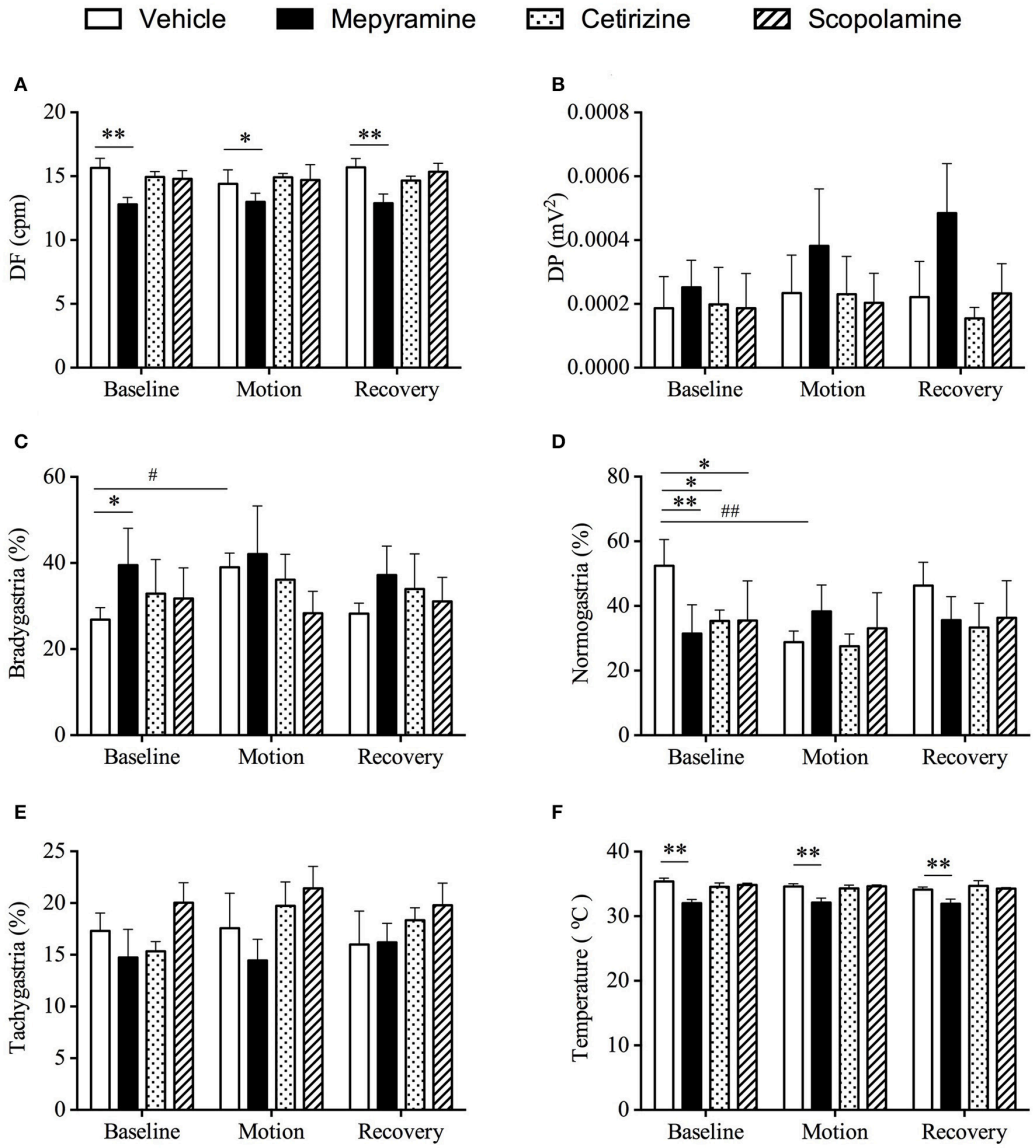
Effect of mepyramine (50 mg/kg), cetirizine (10 mg/kg), and scopolamine (10 mg/kg) on gastric myoelectric activity and core body temperature. **(A)** Dominant frequency (DF); **(B)** Dominant power (DP); **(C)** Bradygastria %; **(D)** Normogastria %; **(E)** Tachygastria %; **(F)** Body temperature. Data represents the mean ± s.e.m. of 6 animals. For inter-group comparison, significant differences compared to vehicle group are shown as *p < 0.05, **p < 0.01 (repeated measures two-way ANOVA followed by Bonferroni tests), ^#^*p* < 0.05, ^##^*p* < 0.01 (repeated measures two-way ANOVA followed by Bonferroni tests) was applied when referring to intra-group comparison. Baseline refers to a 10 min period immediately before provocation motion; recovery indicates 10 min immediately after provocation motion.

**Figure 6:**
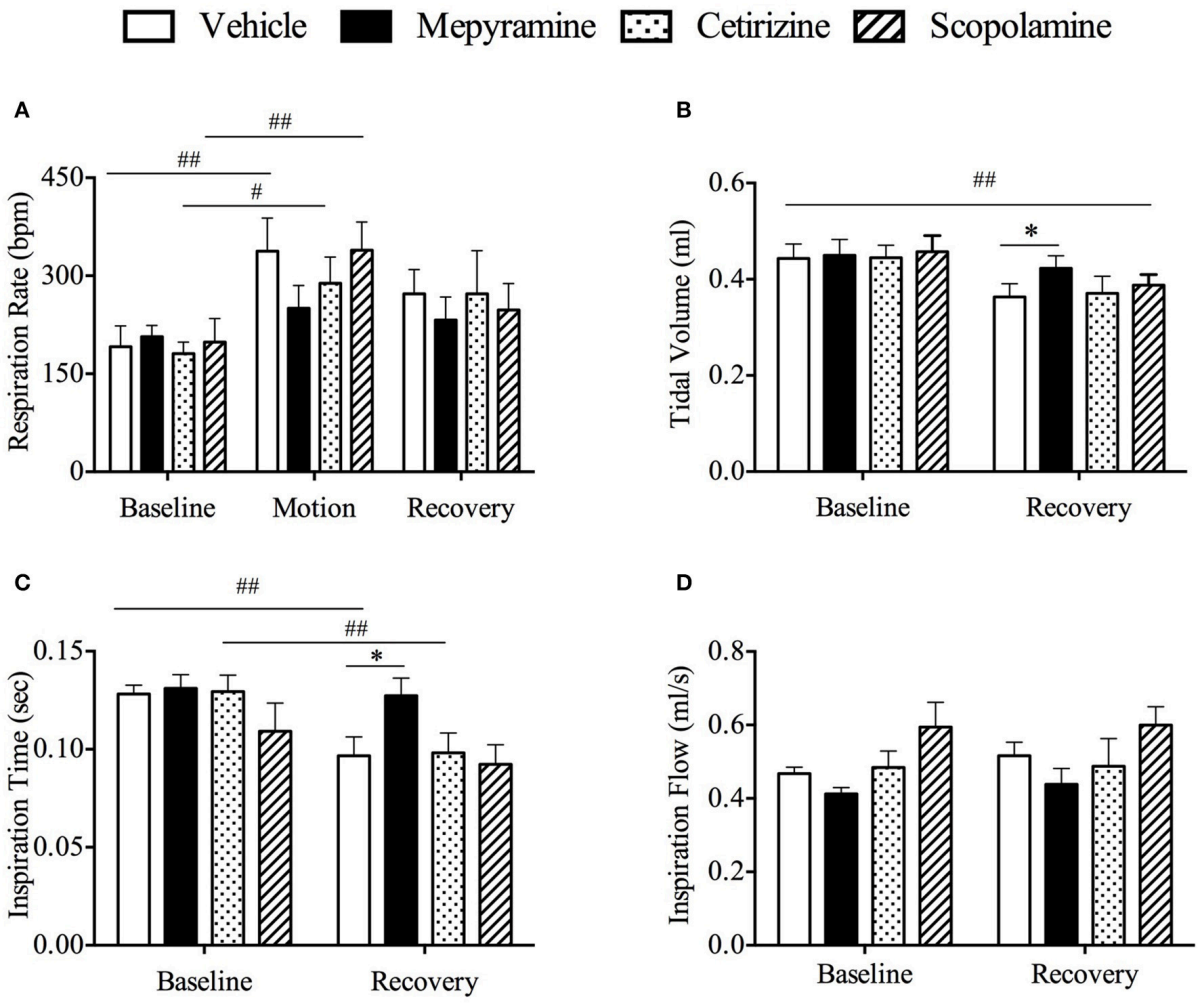
Effect of mepyramine (50 mg/kg), cetirizine (10 mg/kg), and scopolamine (10 mg/kg) on respiratory pattern. **(A)** Respiratory rate; **(B)** Tidal volume; **(C)** Inspiration time; **(D)** Inspiration flow. Data represents the mean ± s.e.m. of 6 animals. For inter-group comparison, significant differences compared to vehicle group are shown as *p < 0.05 (repeated measures two-way ANOVA followed by Bonferroni tests), significant differences compared to baseline are shown as ^#^*p* < 0.05, ^##^*p* < 0.01 (repeated measures two-way ANOVA followed by Bonferroni tests) when referring to intra-group comparison. Baseline refers to a 10 min period immediately before provocation motion; recovery indicates 10 min immediately after provocation motion.

Baseline data for animals prior to randomization was: DF = 15.6 ± 0.2 cpm; DP = 5.7 ± 0.2 ^*^ 10^−4^ mv^2^; and 28.3 ± 1.6% of power was in the bradygastric range; 35.1 ± 2.7% of power was in the normogastric range; and 22.7 ± 1.7% of power was in the tachygastric range (*n* = 24). Only pre-treatment of animals with mepyramine affected the DF, with an 18.4% reduction being recorded (Figure 5A, *p* < 0.01). There was no significant difference among all groups for DP (Figure 5B, *p* > 0.05). The % power in the bradygastric range of the mepyramine-treated animals was also significantly higher than in the vehicle-treated animals (Figure 5C, *p* < 0.05). Consistent with the initial pre-screening study, the % power in the bradygastric range in the vehicle group increased (~50%) significantly during motion when compared with baseline (Figure 5C, *p* < 0.05). Mepyramine-, cetirizine-, and scopolamine-treated animals had a significant lower % of power in the normogastric range in comparison with vehicle group during baseline (Figure 6D, *p* < 0.05). A reduction in the % power of normogastria (~46%) during motion was only observed in vehicle group and is consistent with data obtained during pre-screening (Figure 6D, *p* < 0.01). The % power of tachygastria was not significantly different among the four groups during motion (Figure 6E, *p* > 0.05). With respect to subcutaneous temperature, there was no significant difference among vehicle, cetirizine- and scopolamine- treated groups during the 60 min pre-treatment time. Conversely, mepyramine-caused a substantial fall of ~2.5°C (Figure 6F, *p* < 0.01). A drop of temperature elicited by provocative motion was subsequently observed in vehicle-, cetirizine-, and scopolamine-treated animals, with maximum drops of 0.92 ± 0.2°C, 0.26 ± 0.2°C, and 0.74 ± 0.2°C, respectively (Figure 6F). There was a continuous but slight drop in body temperature during 1 h further recording after motion in scopolamine treated animals, while animals in both vehicle and cetirizine groups remained stable. Body temperature in mepyramine-treated animals gradually returned to normal by the end of the 1 h period. There was no significant difference among the four groups at the endpoint of recording (data not shown).

### Effect of mepyramine, cetirizine, and scopolamine on respiration pattern

The respiratory pattern was essentially the same as observed in the initial screening experiments (above). Basal data prior to randomization was: respiratory rate of 349.9 ± 19.8 bpm; tidal volume was 0.44 ± 0.02 ml; inspiration time was 0.09 ± 0.004 s; and inspiration flow was 0.72 ± 0.03 ml/s (*n* = 24). There was no significant difference in respiration rate among all groups during the baseline period (Figure 6A, *p* > 0.05). However, the respiration rate increased significantly during motion in vehicle, cetirizine and scopolamine, but not in mepyramine treated animals (Figure 6A, *p* > 0.05); the increase was ~42.7% above baseline values (Figure 6A, *p* < 0.01). During the recovery period, there was also a significant reduction in tidal volume in all groups compared with baseline (Figure 6B, *p* < 0.01), but the reduction appeared less for mepyramine group, which remained significantly higher than vehicle group (Figure 6B, *p* < 0.05). In terms of inspiration time, it dramatically decreased during the recovery period in both the vehicle and cetirizine groups compared with baseline (Figure 6C, *p* < 0.01), but not in the mepyramine and scopolamine groups (Figure 6C, *p* > 0.05). Inspiration time in mepyramine group was significantly higher than for the other three groups during recovery (Figure 6C, *p* < 0.05). There was no significant difference in inspiration flow among all groups during the recovery period (Figure 6D, *p* > 0.05).

### Correlations of physiological parameters and MFDFA analysis of GMA

The physiological parameters obtained from the pre-screening study were used to perform correlative analyses during the baseline, motion, and recovery periods, respectively. Episode data correlated positively with respiration rate during motion and recovery with *p*-values of 0.017 and 0.006, respectively (Supplementary Figures 1A,B). Moreover, several positive correlations were also observed between DF and body temperature during baseline, motion and recovery with *p* < 0.0001, 0.007, and 0.0028, respectively (Supplementary Figures 1C–E). A retrospective analysis of data between animals that had vomited and not vomited showed a significant difference in body temperature during baseline, motion and recovery (Supplementary Figures 2A–C, *p* < 0.05; *p* < 0.05; *p* < 0.01, respectively). However, there was no difference in Δ temperature between animals that had vomited and those resistant to motion (Supplementary Figure 2D, *p* > 0.05). In the animals that had emesis, no significant correlation was observed between the number of episodes of emesis and basal body temperature or Δ temperature (data not shown).

MFDFA analysis of GMA did not reveal significant differences for the width of singularity strength Δα of between baseline, motion and recovery periods during the pre-screening study (1.24 ± 0.03 vs. 1.17 ± 0.03 vs. 1.23 ± 0.03, respectively, *p* > 0.05) (Supplementary Figure 3A). A representative multi-fractal spectrum graph is shown in Supplementary Figure 3B. Raw traces of GMA from baseline, motion and recovery periods are also shown in Supplementary Figure 3C. No significant difference in Δα was observed among all the groups during drug/vehicle treatment (Supplementary Table 1).

### Effect of mepyramine, cetirizine, and scopolamine on motion-induced c-Fos expression in brain

**Figure 7:**
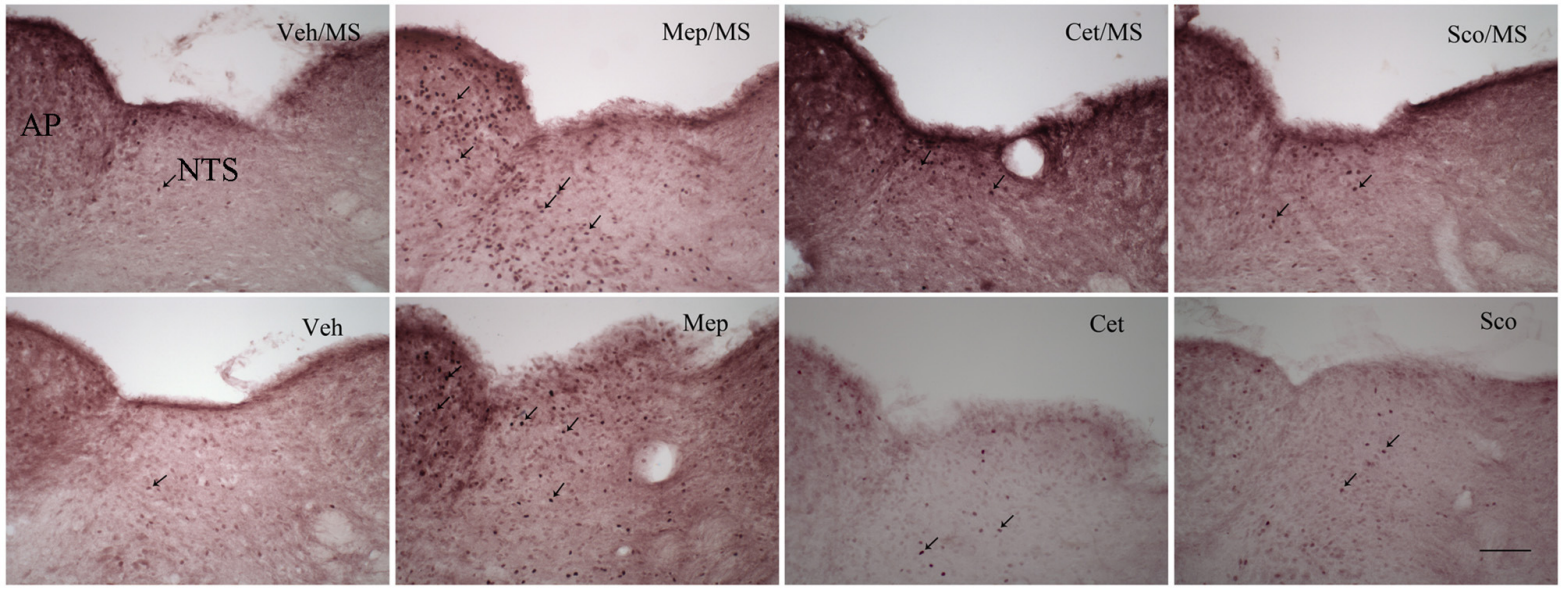
Representative photomicrographs illustrating c-fos expression (violet nuclear label) in the caudal brainstem after administration of saline (2 ml/kg), mepyramine (50 mg/kg), cetirizine (10 mg/kg), and scopolamine (10 mg/kg). Veh/MS, vehicle and motion stimulus; Mep/MS, mepyramine and motion stimulus; Cet/MS, cetirizine and motion stimulus; Sco/MS, scopolamine and motion stimulus; Veh, saline without provocative motion; Mep, mepyramine without provocative motion; Cet, cetirizine without provocative motion; Sco, scopolamine without provocative motion. Arrows show some of the activated c-fos positive cells. AP, area postrema; NTS, nucleus tractus solitarius. Scale bar: 100 μm.

**Figure 8:**
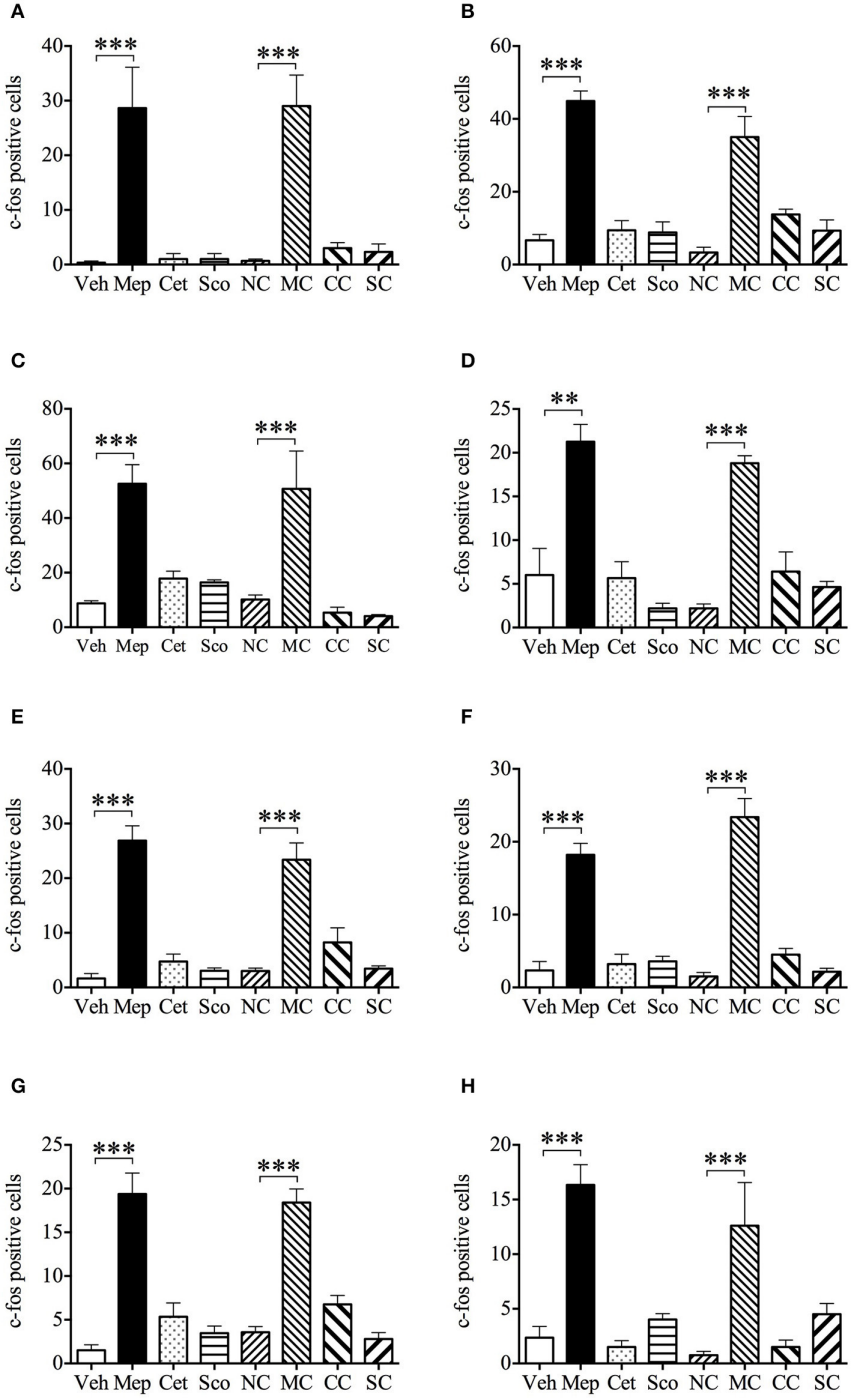
Effect of mepyramine (50 mg/kg), cetirizine (10 mg/kg), and scopolamine (10 mg/kg) on motion-induced c-fos expression in the brain of *Suncus murinus*. Data represents the mean ± s.e.m. of 6 animals. **(A)** Area postrema; **(B)** Nucleus tractus solitarius; **(C)** Medial vestibular nucleus; **(D)** Ventromedial hypothalamic nucleus; **(E)** Dorsomedial hypothalamic nucleus; **(F)** Bednucleus part of lateral hypothalamus; **(G)** Paraventricular hypothalamic nucleus; **(H)** Arcuate hypothalamic nucleus. NC, vehicle control (saline without provocative motion); MC, mepyramine control (mepyramine without provocative motion); CC, cetirizine control (cetirizine without provocative motion); SC, scopolamine control (scopolamine without provocative motion). Significant differences compared to vehicle group or the negative control group are shown as **p < 0.01, ***p < 0.001 (One-way ANOVA followed by Bonferroni tests).

Representative photomicrographs of c-fos staining in the brainstem are shown in Figure 7. In the vehicle group, 10 min of provocative motion itself did not induce c-fos expression in AP, NTS, MVe, VMH, DMH, PLH, PVH, and Arc compared with the negative control of vehicle group (same protocol, but without motion) (Figure 8, *p* > 0.05). However, mepyramine, but not cetirizine or scopolamine, caused a significant increase in c-fos expression in AP, NTS MVe, VMH, DMH, PLH, PVH, and Arc compared with the vehicle group in animals exposed to motion (Figure 8, *p* < 0.001). Mepyramine alone without motion (sham motion conditions) also induced significant increases in c-fos expression in the brain areas that we focussed on (Figure 8, *p* < 0.001). In other brain areas such as ventral and dorsal part of medullary reticular nucleus, the caudal part of spinal trigeminal nucleus, and the hypoglossal nucleus, there were no detectable increase in c-fos, indicating that the effects of mepyramine were not non-specific in nature. Cetirizine and scopolamine alone, without motion, did not cause c-fos expression in the brain.

## Discussion

The present investigation is the first to use radiotelemetry in conjunction with whole body plethysmography to investigate mechanisms of emesis and respiratory function in conscious *Suncus murinus*. This enabled a unique insight into the mechanisms of motion-induced emesis and changes in behavior, gastric myoelectric activity (GMA), and respiration during treatment with brain penetrating (mepyramine) and non-brain penetrating (cetirizine) antihistamines and the muscarinic receptor antagonist, scopolamine. Below we discuss the use of this novel combined approach to recording emesis, reassess the role of histamine and muscarinic receptors in motion sickness, and consider the insights into central emetic pathways provided by the c-fos analysis.

### Baseline respiratory, temperature, and GMA values and the response to motion

Our experimental design permitted a collection of a vast amount of basal data as we prepared to screen for motion sensitivity. Thus, we report for the first time the respiratory parameters of conscious *Suncus murinus* (mean weight ~65 g). In our studies, we also corrected volume measurements for real-time body temperature, which improves accuracy of calculations. *Suncus murinus* had a respiratory frequency of ~243 bpm and a tidal volume of ~0.47 ml. These values are at least twice as high as reported for mice (~20 g) (Mitzner et al., 2001; Mozzini Monteiro et al., 2016). Body temperature (measured subcutaneously) was ~35°C and GMA was typified by a DF of 15 cpm, with 44% of the percentage power being in the normogastric range, which is consistent with our previous studies (Percie du Sert et al., 2010). Behaviorally, the animals were not particularly active when placed in the respiratory recording chamber, spending about 65% of the time resting, with scratching and sniffing predominating as behaviors.

Predictably, motion-induced emesis was easily visible, but we could not confidently score other behaviors when the chamber was moving. However, from the pressure waveforms, we could reliably identify emetic events, with burst analysis showing approximately 6.5 retches/vomits per episode, which is similar to that recorded in other studies of either conscious (6.5 ± 0.2 retches/episode) or anesthetized (7.1 ± 0.7 retches/episode) *Suncus murinus* (Andrews et al., 1996; Huang et al., 2011). We also identified that respiratory rate increased by ~59%, with a concurrent 28% reduction in tidal volume (inspiratory time also reduced) during motion; increases in respiratory rate of the order of about 4–12% are also seen in humans experiencing nausea induced by a simulated roller coaster ride (Gavgani et al., 2016) and in dogs motion-induced emesis may also be accompanied by panting (Crampton, 1990). A recently published clinical study demonstrated that motion also induced an increase of respiratory rate, oxygen consumption and carbon dioxide production, and these effects were more prominent in highly-susceptible participants (Chen et al., 2016). During the recovery period, the respiratory rate remained elevated and the tidal volume remained lower. There was also an increase in scratching activity and a decrease in sniffing. There was a positive correlation between respiratory rate and the number of emetic episodes during motion and the recovery period, but it is not known if this relates more to the stress of the tests, since high respiratory frequency and reduced sniffing is a characteristic of anxiety in rodents (Carnevali et al., 2013). Nevertheless, any potential contribution of an increase in respiratory rate with links to forebrain functioning (e.g., anxiety or “nausea”) needs to be made cautiously since such changes also occur prior to emetic episodes induced by electrical or chemical stimulation of brain pathways and can be seen in anesthetized and decerebrate animals (Bradley et al., 1987; Koga and Fukuda, 1990; Howard and Sears, 1991). As regards GMA, motion induced a reduction in normogastria (Percie du Sert et al., 2010) and there was also a slight fall in body temperature (Ngampramuan et al., 2014; Tu et al., 2017); the latter effect has been documented consistently across several species and has been suggested to be associated with nausea (Nalivaiko et al., 2014). Whilst motion clearly affected the % of power partitioning of GMA, MFDFA revealed that the actual structure of the slow waves themselves did not appear to be disrupted.

### The role histamine and acetylcholine receptors in motion-induced emesis

*Suncus murinus* is an established model for investigation of motion sickness (Ueno et al., 1987, 1988). Previous studies using this species showed that older generation brain penetrant antihistamines, with additional muscarinic receptor blocking activity (e.g., promethazine, diphenhydramine; 20–50 mg/kg), have some activity to reduce (~22% reduction) motion-induced emesis, whereas the H_1_ selective antihistamine, mepyramine (pyrilamine; 20 mg/kg), is less effective (~11%) (Ueno et al., 1988). Comparatively, a more marked effect (50~90% reduction in episodes) is observed following treatment with histamine depleting agents (Kaji et al., 1991), indicating a more prominent role for histamine (possibly mediated via other histamine receptors) than indicated by the antagonist studies. However, it is pertinent that scopolamine was reported previously to have low potency (100 mg/kg) to reduce motion-induced emesis in *Suncus murinus* (Ueno et al., 1988). In view of these anomalies, we considered that the pharmacology of *Suncus murinus* histamine and muscarinic receptors may be atypical and could explain why mepyramine and scopolamine appeared less effective than expected: the *in vitro* studies on the ileum provided some data to support this hypothesis.

Histamine had mixed actions on *Suncus murinus* isolated ilea segments; there was an initial transient relaxation followed by a sustained contraction, which differs from effects observed in other species, such as guinea pigs (for review see Parsons, 1982). The contractile action appeared independent of the cholinergic system (resistant to atropine and hexamethonium), but was partially dependent on enteric nerves, since tetrodotoxin reduced the contractile response by 52%. Both mepyramine and cetirizine behaved as low-potency, non-competitive antagonists, with pK_*b*_ values of 7.5 and 8.4, respectively. On rat and guinea pig H_1_ receptors, both drugs are competitive antagonists, with pA_2_ values of ~9.6 and ~9.4, respectively (Koo, 1983; Suhagia et al., 2006). Conversely, scopolamine behaved as a competitive antagonist, with a pA_2_ of 9.5, consistent with data on rat and human tissues (Brown et al., 1980; Halim et al., 1981). This leads us to conclude that *Suncus murinus* H_1_ receptors differ from those in the rat, guinea, pig and human, whereas muscarinic receptors appear broadly similar.

Taking the above into account, as well as considering the potency of mepyramine and cetirizine from *in vivo* studies (Al Suleimani et al., 2008), we selected doses of mepyramine and scopolamine of 50 and 10 mg/kg, respectively; the choice of the dose of mepyramine being 2.5 times higher than those used in the original motion-induced emesis studies (Ueno et al., 1988). Comparison of the data for scopolamine from *in vivo* studies in rats (Morita et al., 1988; Yu et al., 2007) led us to select a dose of 10 mg/kg, which we considered sufficient to block muscarinic receptors (10 times lower than in the original studies) (Ueno et al., 1988).

### Attenuation of motion-induced effects by blockade of central H_1_ receptors and muscarinic receptors

One of the major findings of the present study is that mepyramine was more effective than cetirizine in preventing motion-induced emesis. This suggests that H_1_ receptors located centrally mediate the anti-emetic effects of antihistamines. Indeed, mepyramine was more active than scopolamine, but this advantage needs to be considered against the backdrop of effects on behavior, temperature homeostasis, and GMA. Mepyramine was the only compound to cause a reduction in scratching behavior and a shift from resting to lying flat, which may indicate sedation. This may also explain why mepyramine appeared to increase inter-retch interval, effectively increasing the duration of individual episodes. During the recovery period, mepyramine appeared to increase inspiratory time and tidal volume, which may or may not be related to its action to cause sedation. Alternatively, this pattern may be interpreted as “deep breathing,” and “deep breathing” techniques are used to abate nausea and emesis in man (Sang et al., 2003; Sites et al., 2014). Yet in our studies, it might not be possible to interpret these data relative to mechanisms controlling nausea, since scopolamine did not share the profile and the situation is complicated further since antihistamines and scopolamine have anxiolytic properties that could impact on the respiratory pattern if it also had a component involving stress (Rodgers and Cole, 1995; Raber, 2005).

It is pertinent that mepyramine was the only treatment to decrease DF and cause marked hypothermia that may have also contributed to its bradygastric action; these effects persisted throughout testing and recovery. An examination of the collective GMA data revealed that all treatments reduced the percentage power of normogastria. Data from isolated murine interstitial cell of Cajal (ICC), show histamine increases the resting membrane potential but not the frequency of slow waves (Kim et al., 2013) and muscarinic receptor agonists have positive chronotropic effects on slow waves *in vitro* and *in vivo* in a number of species (Sanders et al., 2006). No studies have examined isolated ICCs from *Suncus murinus*, but our data suggests that there may be a basal tone on ICC involving histamine and acetylcholine although this requires clarification.

### Novel insights into central emetic mechanisms from c-Fos studies

Our most disappointing finding was that provocative motion did not elicit any detectable activation of neurons in the studied regions of the brain. Our previous studies using *Suncus murinus* have shown that stimuli inducing 6–20 episodes of emesis (average from all studies ~10 episodes) are associated with significant increases of c-fos expression in the brainstem (NTS, AP), hypothalamus and amygdala (Chan et al., 2013, 2014). However, our motion stimulus, which produced ~10 episodes over 10 min, did not. There have only been two other studies where motion-induced emesis has been studied in conjunction with immunohistochemistry for c-fos. In the first study, c-fos expression was readily observed in animals with ~21 episodes when animals were exposed for 30 min (Ito et al., 2003). In the follow up study comparing animals that readily vomited to motion, with those selectively bred to be insensitive, c-fos expression could still be observed in the animals that were less response following the 30 min stimulus (Ito et al., 2005). One of the major differences between our studies and those previously published, therefore, is the duration of the stimulus, and not the number of episodes (Chan et al., 2013, 2014). If this is indeed the case, it is possible that the pattern of c-fos expression seen following longer exposure times to motion does not exclusively relate to emetic mechanisms alone and may include motion-induced changes in blood flow, discomfort and stress or malaise and/or nausea (See Ito et al., 2003, 2005; Yates et al., 2014). Retrospectively, therefore, it may have been better to have used a longer duration stimulus, although interpretation of data arising from such studies should be made cautiously (see Harris, 1998, for a review). Unfortunately, however, none of the previous studies used anti-emetics to qualify the c-fos expression patterns to mechanisms of motion sickness.

Nonetheless, we did identify that mepyramine (1 h pretreatment, with or without motion) could induce large increases in c-fos expression in the AP, NTS MVe, VMH, DMH, PLH, PVH, and Arc, independent of whether the animals experienced emesis or not. This pattern of c-fos activation is paradoxical, being more expected from treatments that induce (e.g., cisplatin, exendin-4, resiniferatoxin), rather than those that inhibit emesis; anti-emetics to date have been shown to only decrease such increases (Andrews et al., 2000; De Jonghe and Horn, 2009; Chan et al., 2013). Certainly, scopolamine did not share this profile of activation and yet it was only slightly less effective at inhibiting motion-induced emesis.

In order to propose a possible explanation for the pattern of c-fos induced by mepyramine, we need to consider that it had pharmacological effects different from cetirizine and scopolamine (mepyramine caused changes in behavior that we interpreted as inhibitory or sedative, and there was also clear hypothermia and a reduced DF in the GMA recorded from the gastric antrum). Clearly, there may have been other physiological changes that our studies were not designed to detect (e.g., blood pressure). Next, we need consider several aspects of histaminergic pathways and signaling. The cell bodies of histaminergic neurons are located in the posterior region of the lateral hypothalamus (in the tuberomammillary nucleus) and send projections toward other parts of the hypothalamus, cortex, hippocampus, amygdala and also toward the brainstem (Karasawa et al., 2001), although another major source of histamine in the brain is from mast cells (Goldschmidt et al., 1985; Kaji et al., 1991); the relative contribution of histamine in emetic mechanisms coming from neurons vs. mast cells is not known (Lucot and Takeda, 1992). H_1_ receptors are located in many brain areas, cerebral cortex, limbic system, NTS, and dorsal motor nucleus of the vagus nerve (Palacios et al., 1981; Martinez-Mir et al., 1990). Though G_*q*/11_ proteins, these receptors are coupled to the phospholipase C, which in turn induces calcium-dependent events and excitation of target cells (Banu and Watanabe, 1999).

If we follow the previous discoveries made in *Suncus murinus* and in cats, we may conclude that the brainstem NTS and lateral reticular formation, including the ventrolateral reticular formation, the inferior olive, and vestibular nuclei and nucleus ambiguous are involved. Evidence from physiological experiments indicates that NTS neurons respond to electrical stimulation of the VIIIth cranial nerve which links labyrinthine receptors to the NTS, relaying to the pattern generator for emesis in the reticular formation (See Ito et al., 2003, 2005; Yates et al., 2014). Unfortunately, there have been no studies where intracerebral administration of histamine receptor antagonists have been made into any of these nuclei during motion-induced emesis experimentation. However, it is thought that during provocative motion, a neural mismatch signal activates histaminergic neurons in the hypothalamus, and the histaminergic descending pathway to potentially stimulate H_1_ receptors in the brainstem's “emetic center” (Takeda et al., 1986; Horii et al., 1993; Uno et al., 1997; Schmäl, 2013). Whilst the precise role of neuron and mast cell sources of histamine are not known, it is known that H_1_ receptors are densely located in the NTS and DMNV, and also the vestibular nucleus; all areas are behind the blood brain barrier. It is conceivable that receptors in the brainstem are key to the anti-emetic mechanism of action of the anti-histamines, particularly since our burst analysis showed that only mepyramine altered emetic patterns. There is also good evidence that histamine administered into the 4th ventricle is more potent to induce emesis than following administration into the lateral ventricle in dogs which tends to implicate brainstem mechanisms; the latency to induce emesis is also shorter and licking, tachypnoea, restlessness, was also observed (Bhargava and Dixit, 1968); mepyramine given into the lateral ventricles antagonized the emesis induced by histamine given via the same route (Bhargava et al., 1976). Electrophysiologically, there are studies showing that histamine can induce and enhance spontaneous firing of neurons in the vestibular nucleus in rats, effects that are reduced by mepyramine and the H_2_ receptor antagonist, cimetidine. However, mepyramine alone did not appear to have any effect to depolarize or hyperpolarize neurons, or to change the frequency of spontaneous firing (Yu et al., 2015). In another rat study, histamine reportedly induced firing of neurons in the NTS, which was blocked by the H_1_ receptor antagonist, triprolidine. However, triprolidine failed to induce significant changes when tested alone (Poole et al., 2008).

Although our functional studies on isolated ileal segments in *Suncus* revealed all of our compounds as antagonists, antihistamines have been documented to possess inverse agonist properties at H_1_ receptors, and mepyramine is a proven full inverse agonist (Fitzsimons et al., 2004). Does this mean, therefore, that there are constitutively active H_1_ receptors in the brain that mepyramine can exert negative efficacy to transduce c-fos? Histamine itself can trigger expression of c-fos through PKCα, MEK-1, and MAP kinase (Megson et al., 2001), but 5-HT_2C_ receptors, which are also coupled to G_q/11_ do in fact increase c-fos expression in the brain following treatment with inverse agonists (Navailles et al., 2013). Clearly, if this is also the case for mepyramine, it would alter our concept of how antihistamines are acting to reduce emesis and the possibility that there are differences in constitutively active H_1_ receptors between motion-sensitive and non-sensitive individuals. Constitutive activity is related to receptor density and the level of G protein expression and upregulation of H_1_ receptor expression levels has been found in patients with allergic rhinitis (Dinh et al., 2005). It may be that motion-sensitive individuals have a higher density of H_1_ receptors in emetic circuits, and that these patients benefit the most from the use of H_1_ anti-histamines. It is certainly difficult to reconcile any hypothesis from our limited data, and there is a possibility that the effects we have observed are secondary to mepyramine to increase inhibition within emetic circuits. However, this latter hypothesis is less likely, as antihistamines do not have broad inhibitory effects.

Both brain penetrating H_1_ antihistamines and scopolamine are noted to reduce motion-induced nausea in man (see introduction), but our studies were designed to primarily study emesis. The behavioral and physiological readouts that we obtained therefore cannot be considered representative of nausea alone. Nevertheless, we compared all the data to see if there was any evidence that mepyramine and scopolamine had mechanisms in common that could reflect an ability to reduce “nausea” (see Introduction); certainly nothing was evident from the pattern of c-fos. The only variable, apart from emesis, that was consistently modulated by both mepyramine and scopolamine was on GMA where a common reduction in the % power of bradygastria was seen. However, cetirizine also had the same action and during motion mepyramine and scopolamine failed to differentiate from cetirizine. It is possible that this pattern relates to the action of the compounds directly on the ICCs themselves. Studies therefore need to be conducted where the anti-emetics are administered centrally, to avoid exposure on the ICC, permitting a more confident interpretation of mechanisms relative to nausea and emesis.

### Conclusions and perspectives

In conclusion, we have shown that mepyramine is more effective than cetirizine in preventing motion-induced emesis, indicating that centrally located H_1_ receptors are critically involved. Mepyramine caused behavior indicative of sedation, hypothermia, and a fall in DF in the GMA, whereas cetirizine and the muscarinic receptor antagonists, scopolamine, which was anti-emetic, did not. The effects of mepyramine on respiration and inter-retch/vomit episodes are more difficult to ascribe to the anti-emetic mechanism of action, and requires further clarification. The ability of mepyramine to increase c-fos in the brain to a pattern reminiscent of emetic challenges (but without induction of emesis) provides an important new insight into mechanisms involved in emesis control in general.

## Author contributions

LT, KD, CS, and SC conducted organ bath experiment. LT and ZL performed the animal surgery. LT performed immunostaining, data analysis, and drafted the manuscript. JR, PA, EN, and ZL finalized the manuscript. All authors reviewed the final version of the manuscript and approved submission.

### Conflict of interest statement

The authors declare that the research was conducted in the absence of any commercial or financial relationships that could be construed as a potential conflict of interest.

## Supplementary material

Supplementary Figure 1 Correlations of physiological parameters from pre-screening study. **(A)** Episodes vs. respiratory rate during motion; **(B)** Episodes vs. respiratory rate during recovery; **(C)** DF vs. core body temperature during baseline; **(D)** DF vs. core body temperature during motion; **(E)** DF vs. core body temperature during recovery. Data represents the mean ± s.e.m. of 40–52 animals.

Supplementary Figure 2 Temperature difference between vomited and no-vomited animals during baseline, motion and recovery in pre-screening study. **(A)** Baseline; **(B)** Motion; **(C)** Recovery; **(D)** Δ temperature during motion. Data represents the mean ± s.e.m. of 51 vomited animals and 7 non-vomited animals. Significant differences are shown as ^*^*p* < 0.05, ^**^*p* < 0.01 (unpaired *t*-test).

Supplementary Figure 3 Singularity spectra of time series of GMA that were estimated by multifractal detrended fluctuation analysis (MFDFA). **(A)** The width of singularity strength Δα of baseline, motion, and recovery during pre-screening study; **(B)** Graphs represents *f (*α*)* vs. singularity strength α; **(C)** representative raw traces of gastric slow waves from baseline, motion and recovery. Data represents the mean ± s.e.m. of 40–52 animals.

Supplementary Table 1 Effect of mepyramine (50 mg/kg), cetirizine (10 mg/kg), and scopolamine (10 mg/kg) on the width of singularity strength Δα of GMA. Data represents the mean ± s.e.m. of 6 animals.

## References

[B1] Al Suleimani Y. M. Dong Y. Walker M. J. (2008). Differential responses to various classes of drugs in a model of allergic rhinitis in guinea pigs. Pulm. Pharmacol. Ther. 21, 340–348. 10.1016/j.pupt.2007.08.004 17905620

[B2] Andrews P. Okada F. Woods A. Hagiwara H. Kakaimoto S. Toyoda M.. (2000). The emetic and anti-emetic effects of the capsaicin analogue resiniferatoxin in *Suncus murinus*, the house musk shrew. Br. J. Pharmacol. 130, 1247–1254. 10.1038/sj.bjp.0703428 10903962 PMC1572188

[B3] Andrews P. Torii Y. Saito H. Matsuki N. (1996). The pharmacology of the emetic response to upper gastrointestinal tract stimulation in *Suncus murinus*. Eur. J. Pharmacol. 307, 305–313. 10.1016/0014-2999(96)00275-0 8836619

[B4] Banu Y. Watanabe T. (1999). Augmentation of antigen receptor–mediated responses by histamine H_1_ receptor signaling. J. Exp. Med. 189, 673–682. 10.1084/jem.189.4.673 9989982 PMC2192933

[B5] Benavides J. Schoemaker H. Dana C. Claustre Y. Delahaye M. Prouteau M.. (1995). *In vivo* and *in vitro* interaction of the novel selective histamine H_1_ receptor antagonist mizolastine with H_1_ receptors in the rodent. Arzneimittelforschung 45, 551–558. 7612054

[B6] Bertolini G. Straumann D. (2016). Moving in a moving world: a review on vestibular motion sickness. Front. Neurol. 7:14. 10.3389/fneur.2016.00014 26913019 PMC4753518

[B7] Bhargava K. Dixit K. (1968). Role of the chemoreceptor trigger zone in histamine-induced emesis. Br. J. Pharmacol. 34, 508–513. 10.1111/j.1476-5381.1968.tb08479.x 4387255 PMC1703476

[B8] Bhargava K. Dixit K. Palit G. (1976). Nature of histamine receptors in the emetic chemoreceptor trigger zone. Br. J. Pharmacol. 57, 211–213. 10.1111/j.1476-5381.1976.tb07469.x 938795 PMC1667111

[B9] Bradley D. Pascoe J. Paton J. Spyer K. (1987). Cardiovascular and respiratory responses evoked from the posterior cerebellar cortex and fastigial nucleus in the cat. J. Physiol. 393, 107–121. 10.1113/jphysiol.1987.sp016813 3446792 PMC1192383

[B10] Brown D. Forward A. Marsh S. (1980). Antagonist discrimination between ganglionic and ileal muscarinic receptors. Br. J. Pharmacol. 71, 362–364. 10.1111/j.1476-5381.1980.tb10948.x 7470751 PMC2044477

[B11] Carnevali L. Trombini M. Rossi S. Graiani G. Manghi M. Koolhaas J. M.. (2013). Structural and electrical myocardial remodeling in a rodent model of depression. Psychosom. Med. 75, 42–51. 10.1097/PSY.0b013e318276cb0d 23257930

[B12] Chan S. W. He J. Lin G. Rudd J. A. Yamamoto K. (2007). Action of GLP-1 (7-36) amide and exendin-4 on *Suncus murinus* (house musk shrew) isolated ileum. Eur. J. Pharmacol. 566, 185–191. 10.1016/j.ejphar.2007.03.050 17475239

[B13] Chan S. W. Lin G. Yew D. T. W. Yeung C. K. Rudd J. A. (2013). Separation of emetic and anorexic responses of exendin-4, a GLP-1 receptor agonist in *Suncus murinus* (house musk shrew). Neuropharmacology 70, 141–147. 10.1016/j.neuropharm.2013.01.013 23357334

[B14] Chan S. W. Lu Z. Lin G. Yew D. T. W. Yeung C. K. Rudd J. A. (2014). The differential antiemetic properties of GLP-1 receptor antagonist, exendin (9–39) in *Suncus murinus* (house musk shrew). Neuropharmacology 83, 71–78. 10.1016/j.neuropharm.2014.03.016 24726308

[B15] Chen C. (2008). Physicochemical, pharmacological and pharmacokinetic properties of the zwitterionic antihistamines cetirizine and levocetirizine. Curr. Med. Chem. 15, 2173–2191. 10.2174/092986708785747625 18781943

[B16] Chen C. L. Li P. C. Chuang C. C. Lung C. W. Tang J. S. (2016). Comparison of motion sickness-induced cardiorespiratory responses between susceptible and non-susceptible subjects and the factors associated with symptom severity, in 2016 IEEE 16th International Conference on Bioinformatics and Bioengineering (BIBE) (Taiwan).

[B17] Cheung B. S. Heskin R. Hofer K. D. (2003). Failure of cetirizine and fexofenadine to prevent motion sickness. Ann. Pharmacother. 37, 173–177. 10.1177/106002800303700201 12549941

[B18] Cowings P. S. Suter S. Toscano W. B. Kamiya J. Naifeh K. (1986). General autonomic components of motion sickness. Psychophysiology 23, 542–551. 10.1111/j.1469-8986.1986.tb00671.x 3809361

[B19] Crampton G. H. (1990). Motion and Space Sickness. Florida: CRC Press.

[B20] De Jonghe B. C. Horn C. C. (2009). Chemotherapy agent cisplatin induces 48-h Fos expression in the brain of a vomiting species, the house musk shrew (*Suncus murinus*). Am. J. Physiol. Regul. Integr. Comp. Physiol. 296, R902–R911. 10.1152/ajpregu.90952.2008 19225146 PMC2698611

[B21] Dinh Q. Cryer A. Dinh S. Peiser C. Wu S. Springer J.. (2005). Transcriptional up-regulation of histamine receptor-1 in epithelial, mucus and inflammatory cells in perennial allergic rhinitis. Clin. Exp. Allergy 35, 1443–1448. 10.1111/j.1365-2222.2005.02359.x 16297140

[B22] Fitzsimons C. P. Monczor F. Fernández N. Shayo C. Davio C. (2004). Mepyramine, a histamine H_1_ receptor inverse agonist, binds preferentially to a G protein-coupled form of the receptor and sequesters G protein. J. Biol. Chem. 279, 34431–34439. 10.1074/jbc.M400738200 15192105

[B23] Gaddum J. Hameed K. A. Hathway D. Stephens F. (1955). Quantitative studies of antagonists for 5-hydroxytryptamine. Q. J. Exp. Physiol. Cogn. Med. Sci. 40, 49–74. 10.1113/expphysiol.1955.sp001097 14371990

[B24] Gavgani A. M. Nesbitt K. V. Blackmore K. L. Nalivaiko E. (2016). Profiling subjective symptoms and autonomic changes associated with cybersickness. Auton. Neurosci. 203, 41–50. 10.1016/j.autneu.2016.12.004 28010995

[B25] Golding J. F. (2006). Motion sickness susceptibility. Auton. Neurosci. 129, 67–76. 10.1016/j.autneu.2006.07.019 16931173

[B26] Golding J. F. Gresty M. A. (2015). Pathophysiology and treatment of motion sickness. Curr. Opin. Neurol. 28, 83–88. 10.1097/WCO.0000000000000163 25502048

[B27] Goldschmidt R. C. Hough L. B. Glick S. D. (1985). Rat brain mast cells: contribution to brain histamine levels. J. Neurochem. 44, 1943–1947. 10.1111/j.1471-4159.1985.tb07191.x 3989570

[B28] Halim S. Kilbinger H. Wessler I. (1981). Pirenzepine does not discriminate between pre-and postsynaptic muscarine receptors in the guinea-pig small intestine. Scand. J. Gastroenterol. Suppl. 72, 87–94. 6958001

[B29] Harris J. A. (1998). Using c-fos as a neural marker of pain. Brain. Res. Bull. 45, 1–8. 10.1016/S0361-9230(97)00277-3 9434195

[B30] Himi N. Koga T. Nakamura E. Kobashi M. Yamane M. Tsujioka K. (2004). Differences in autonomic responses between subjects with and without nausea while watching an irregularly oscillating video. Auton. Neurosci. 116, 46–53. 10.1016/j.autneu.2004.08.008 15556837

[B31] Horii A. Takeda N. Matsunaga T. Yamatodani A. Mochizuki T. Okakura-Mochizuki K.. (1993). Effect of unilateral vestibular stimulation on histamine release from the hypothalamus of rats *in vivo*. J. Neurophysiol. 70, 1822–1826. 8294957 10.1152/jn.1993.70.5.1822

[B32] Horn C. C. Henry S. Meyers K. Magnusson M. S. (2011). Behavioral patterns associated with chemotherapy-induced emesis: a potential signature for nausea in musk shrews. Front. Neurosci. 5:88. 10.3389/fnins.2011.00088 21808604 PMC3139242

[B33] Horn C. C. Wang H. Estival L. Meyers K. Magnusson M. S. (2013). Novel dynamic measures of emetic behavior in musk shrews. Auton. Neurosci. 179, 60–67. 10.1016/j.autneu.2013.07.006 23953843 PMC3844068

[B34] Horn C. Zirpel L. Sciullo M. Rosenberg D. (2016). Impact of electrical stimulation of the stomach on gastric distension-induced emesis in the musk shrew. Neurogastroenterol. Motil. 28, 1217–1232. 10.1111/nmo.12821 27072787 PMC4956516

[B35] Howard R. Sears T. (1991). The effects of opiates on the respiratory activity of thoracic motoneurones in the anaesthetized and decerebrate rabbit. J. Physiol. 437, 181–199. 10.1113/jphysiol.1991.sp018590 1890632 PMC1180042

[B36] Huang D. Meyers K. Henry S. De La Torre F. Horn C. C. (2011). Computerized detection and analysis of cancer chemotherapy-induced emesis in a small animal model, musk shrew. J. Neurosci. Methods. 197, 249–258. 10.1016/j.jneumeth.2011.02.032 21392533 PMC3319687

[B37] Ihlen E. A. (2012). Introduction to multifractal detrended fluctuation analysis in Matlab. Front. Physiol. 3:141. 10.3389/fphys.2012.00141 22675302 PMC3366552

[B38] Ito H. Nishibayashi M. Kawabata K. Maeda S. Seki M. Ebukuro S. (2003). Induction of Fos protein in neurons in the medulla oblongata after motion- and X-irradiation-induced emesis in musk shrews (*Suncus murinus*). Auton. Neurosci. 107, 1–8. 10.1016/S1566-0702(03)00026-2 12927221

[B39] Ito H. Nishibayashi M. Maeda S. Ebukuro S. (2005). Emetic response and neural activity in young musk shrews during the breast-feeding/weaning period: comparison between the high and low emetic response strains using a shaking stimulus. Exp. Anim. 54, 301–307. 10.1538/expanim.54.301 16093643

[B40] Kaji T. Saito H. Ueno S. Yasuhara T. Nakajima T. Matsuki N. (1991). Role of histamine in motion sickness in *Suncus murinus*. Aviation. Space. Environ. Med. 62, 1054–1058. 1741719

[B41] Kantelhardt J. W. Zschiegner S. A. Koscielny-Bunde E. Havlin S. Bunde A. Stanley H. E. (2002). Multifractal detrended fluctuation analysis of nonstationary time series. Phys. A Stat. Mech. Appl. 316, 87–114. 10.1016/S0378-4371(02)01383-3

[B42] Karasawa N. Isomura G. Yamada K. Arai R. Takeuchi T. Nagatsu I. (2001). Distribution of histamine-containing neurons in the laboratory shrew (*Suncus murinus*) brain. Acta Histochem. Cytochem. 34, 431–439. 10.1267/ahc.34.431

[B43] Kim B. J. Kwon Y. K. Kim E. So I. (2013). Effects of histamine on cultured interstitial cells of cajal in murine small intestine. Korean J. Physiol. Pharmacol. 17, 149–156. 10.4196/kjpp.2013.17.2.149 23626477 PMC3634092

[B44] Koga T. Fukuda H. (1990). Characteristic behavior of the respiratory muscles, esophagus, and external anl and urethral sphincters during straining, retching, and vomiting in the decerebrate dog. Jpn. J. Physiol. 40, 789–807. 10.2170/jjphysiol.40.789 1965598

[B45] Kohl R. L. Calkins D. S. Robinson R. E. (1991). Control of nausea and autonomic dysfunction with terfenadine, a peripherally acting antihistamine. Aviat. Space Environ. Med. 62, 392–396. 1675849

[B46] Koo A. (1983). *In vivo* characterization of histamine H_1_- and H_2_-receptors in the rat stomach microcirculation. Br. J. Pharmacol. 78, 181–189. 10.1111/j.1476-5381.1983.tb09379.x 6218851 PMC2044775

[B47] Lucot J. B. Takeda N. (1992). α-fluoromethylhistidine but not diphenhydramine prevents motion-induced emesis in the cat. Am. J. Otolaryngol. 13, 176–180. 10.1016/0196-0709(92)90119-E 1626619

[B48] Martinez-Mir M. Pollard H. Moreau J. Arrang J. Ruat M. Traiffort E.. (1990). Three histamine receptors (H1, H2 and H3) visualized in the brain of human and non-human primates. Brain Res. 526, 322–327. 10.1016/0006-8993(90)91240-H 1979518

[B49] Matsuoka I. Ito J. Takahashi H. Sasa M. Takaori S. (1983). Experimental vestibular pharmacology: a minireview with special reference to neuroactive substances and antivertigo drugs. Acta. Otolaryngol. Suppl. 419, 62–70. 6399658

[B50] Megson A. Walker E. Hill S. (2001). Role of protein kinase Cα in signaling from the histamine H_1_ receptor to the nucleus. Mol. Pharmacol. 59, 1012–1021. 10.1124/mol.59.5.1012 11306682

[B51] Mitzner W. Brown R. Lee W. (2001). *In vivo* measurement of lung volumes in mice. Physiol. Genomics 4, 215–221. 11161000 10.1152/physiolgenomics.2001.4.3.215

[B52] Morita M. Takeda N. Kubo T. Yamatodani A. Wada H. Matsunaga T. (1988). Effects of anti-motion sickness drugs on motion sickness in rats. ORL J. Otorhinolaryngol. Relat. Spec. 50, 330–333. 10.1159/000276008 3186231

[B53] Mozzini Monteiro T. Ferrera Costa H. Carvalho Vieira G. Rodrigues Salgado P. R. Da Silva Stiebbe Salvadori M. G. De Almeida R. N.. (2016). Anti-asthmatic and anxiolytic effects of *Herissantia tiubae*, a Brazilian medicinal plant. Immun. Inflamm. Dis. 4, 201–212. 10.1002/iid3.107 27957328 PMC4879466

[B54] Murdin L. Golding J. Bronstein A. (2011). Managing motion sickness. BMJ 343:d7430. 10.1136/bmj.d7430 22138695

[B55] Nakayama H. Yamakuni H. Higaki M. Ishikawa H. Imazumi K. Matsuo M.. (2005). Antiemetic activity of FK1052, a 5-HT_3_- and 5-HT_4_-receptor antagonist, in *Suncus murinus* and ferrets. J. Pharmacol. Sci. 98, 396–403. 10.1254/jphs.FPJ05001X 16079468

[B56] Nalivaiko E. Davis S. L. Blackmore K. L. Vakulin A. Nesbitt K. V. (2015). Cybersickness provoked by head-mounted display affects cutaneous vascular tone, heart rate and reaction time. Physiol. Behav. 151, 583–590. 10.1016/j.physbeh.2015.08.043 26340855

[B57] Nalivaiko E. Rudd J. A. So R. H. (2014). Motion sickness, nausea and thermoregulation: the “toxic” hypothesis. Temperature 1, 164–171. 10.4161/23328940.2014.982047 27626043 PMC5008705

[B58] Navailles S. Lagière M. Le Moine C. De Deurwaerdere P. (2013). Role of 5-HT_2*C*_ receptors in the enhancement of c-Fos expression induced by a 5-HT_2*B*/2*C*_ inverse agonist and 5-HT_2_ agonists in the rat basal ganglia. Exp. Brain. Res. 230, 525–535. 10.1007/s00221-013-3562-9 23681297

[B59] Ngampramuan S. Cerri M. Del Vecchio F. Corrigan J. J. Kamphee A. Dragic A. S.. (2014). Thermoregulatory correlates of nausea in rats and musk shrews. Oncotarget 5, 1565–1575. 10.18632/oncotarget.1732 24728971 PMC4039232

[B60] Oman C. M. (2012). Are evolutionary hypotheses for motion sickness “just-so” stories? J. Vestib. Res. 22, 117–127. 10.3233/VES-2011-0432 23000611

[B61] Oman C. M. Cullen K. E. (2014). Brainstem processing of vestibular sensory exafference: implications for motion sickness etiology. Exp. Brain. Res. 232, 2483–2492. 10.1007/s00221-014-3973-2 24838552 PMC4130651

[B62] Palacios J. Wamsley J. Kuhar M. (1981). The distribution of histamine H_1_-receptors in the rat brain: an autoradiographic study. Neuroscience. 6, 15–37. 10.1016/0306-4522(81)90240-2 6111763

[B63] Parsons M. (1982). Histamine receptors in alimentary and genito-urinary smooth muscle, in Pharmacology of Histamine Receptors, eds Ganellin C. R. Parsons M. E. (Brisol: Wright PSG), 323–350.

[B64] Percie du Sert N. Chu K. M. Wai M. K. Rudd J. A. Andrews P. L. (2010). Telemetry in a motion-sickness model implicates the abdominal vagus in motion-induced gastric dysrhythmia. Exp. Physiol. 95, 768–773. 10.1113/expphysiol.2009.052001 20360423

[B65] Poole S. L. Lewis D. I. Deuchars S. A. (2008). Histamine depolarizes neurons in the dorsal vagal complex. Neurosci. Lett. 432, 19–24. 10.1016/j.neulet.2007.11.055 18162318

[B66] Raber J. (2005). Histamine receptors as potential therapeutic targets to treat anxiety and depression. Drug Dev. Res. 65, 126–132. 10.1002/ddr.20015

[B67] Reason J. (1978). Motion sickness adaptation: a neural mismatch model. J. R. Soc. Med. 71, 819–829. 731645 10.1177/014107687807101109 PMC1436193

[B68] Reason J. T. Brand J. J. (1975). Motion Sickness. Oxford: Academic press.

[B69] Rodgers R. Cole J. (1995). Effects of scopolamine and its quaternary analogue in the murine elevated plus-maze test of anxiety. Behav. Pharmacol. 6, 283–289. 11224337

[B70] Rudd J. A. Ngan M. P. Wai M. K. (1999). Inhibition of emesis by tachykinin NK_1_ receptor antagonists in *Suncus murinus* (house musk shrew). Eur. J. Pharmacol. 366, 243–252. 10.1016/S0014-2999(98)00920-0 10082206

[B71] Sanders K. M. Koh S. D. Ward S. M. (2006). Interstitial cells of Cajal as pacemakers in the gastrointestinal tract. Annu. Rev. Physiol. 68, 307–343. 10.1146/annurev.physiol.68.040504.094718 16460275

[B72] Sang F. D. Billar J. P. Golding J. F. Gresty M. A. (2003). Behavioral methods of alleviating motion sickness: effectiveness of controlled breathing and a music audiotape. J. Travel Med. 10, 108–111. 10.2310/7060.2003.31768 12650654

[B73] Schild H. (1947). pA, a new scale for the measurement of drug antagonism. Br. J. Pharmacol. 2, 189–206. 10.1111/j.1476-5381.1947.tb00336.x 20258355 PMC1509780

[B74] Schmäl F. (2013). Neuronal mechanisms and the treatment of motion sickness. Pharmacology 91, 229–241. 10.1159/000350185 23615033

[B75] Sharma K. (1997). Prevalence and correlates of susceptibility to motion sickness. Acta Genet. Med. Gemellol. 46, 105–121. 10.1017/S0001566000000660 9492893

[B76] Shupak A. Gordon C. R. (2006). Motion sickness: advances in pathogenesis, prediction, prevention, and treatment. Aviat. Space. Environ. Med. 77, 1213–1223. 17183916

[B77] Simon F. E. R. Simons K. J. (2008). H_1_antihistamines: current status and future directions. World Allergy Organ. J. 1, 145–155. 10.1097/WOX.0b013e318186fb3a 23282578 PMC3650962

[B78] Sites D. S. Johnson N. T. Miller J. A. Torbush P. H. Hardin J. S. Knowles S. S. (2014). Controlled breathing with or without peppermint aromatherapy for postoperative nausea and/or vomiting symptom relief: a randomized controlled trial. J. Perianesth. Nurs. 29, 12–19. 10.1016/j.jopan.2013.09.008 24461278

[B79] Soto E. Vega R. (2010). Neuropharmacology of vestibular system disorders. Curr. Neuropharmacol. 8, 26–40. 10.2174/157015910790909511 20808544 PMC2866460

[B80] Spinks A. Wasiak J. (2011). Scopolamine (hyoscine) for preventing and treating motion sickness. Cochrane Database Syst. Rev. CD002851. 10.1002/14651858.CD002851.pub4 PMC7138049 21678338

[B81] Stern R. M. Koch K. L. Stewart W. R. Lindblad I. M. (1987). Spectral analysis of tachygastria recorded during motion sickness. Gastroenterology 92, 92–97. 10.1016/0016-5085(87)90843-2 3781204

[B82] Suhagia B. N. Chhabria M. T. Makwana A. G. (2006). Design, synthesis and pharmacological screening of a series of N^1^-(substituted) aryl-5, 7-dimethyl-2-(substituted) pyrido (2, 3-d) pyrimidin-4 (3H)-ones as potential histamine H_1_-receptor antagonists. J. Enzyme Inhib. Med. Chem. 21, 681–691. 10.1080/14756360600851104 17252940

[B83] Takeda N. Morita M. Kubo T. Yamatodani A. Watanabe T. Wada H.. (1986). Histaminergic mechanism of motion sickness neurochemical and neuropharmacological studies in rats. Acta Otolaryngol. 101, 416–421. 10.3109/00016488609108626 3727976

[B84] Tashiro N. Kataoka M. Ozawa K. Ikeda T. (2007). The evaluation of whole-body plethysmography as a semiautomated method for analysis of emesis in the house musk shrew (*Suncus murinus*). J. Am. Assoc. Lab. Anim. Sci. 46, 81–85. 17343358

[B85] Tu L. Poppi L. Rudd J. Cresswell E. T. Smith D. W. Brichta A.. (2017). α-9 nicotinic acetylcholine receptors mediate hypothermic responses elicited by provocative motion in mice. Physiol. Behav. 174, 114–119. 10.1016/j.physbeh.2017.03.012 28302571

[B86] Uchino M. Ishii K. Kuwahara M. Ebukuro S. Tsubone H. (2002). Role of the autonomic nervous system in emetic and cardiovascular responses in Suncus murinus. Auton. Neurosci. 100, 32–40. 10.1016/S1566-0702(02)00141-8 12422958

[B87] Ueno S. Matsuki N. Saito H. (1987). *Suncus murinus*: a new experimental model in emesis research. Life Sci. 41, 513–518. 10.1016/0024-3205(87)90229-3 3600192

[B88] Ueno S. Matsuki N. Saito H. (1988). *Suncus murinus* as a new experimental model for motion sickness. Life Sci. 43, 413–420. 10.1016/0024-3205(88)90520-6 2899827

[B89] Uno A. Takeda N. Horii A. Morita M. Yamamoto Y. Yamatodani A.. (1997). Histamine release from the hypothalamus induced by gravity change in rats and space motion sickness. Physiol. Behav. 61, 883–887. 10.1016/S0031-9384(96)00613-0 9177562

[B90] Yates B. J. Catanzaro M. F. Miller D. J. McCall A. A. (2014). Integration of vestibular and emetic gastrointestinal signals that produce nausea and vomiting: potential contributions to motion sickness. Exp. Brain Res. 232, 2455–2469. 10.1007/s00221-014-3937-6 24736862 PMC4112154

[B91] Yu M. Li P. Basnet S. K. Kumarasiri M. Diab S. Teo T.. (2015). Discovery of 4-(dihydropyridinon-3-yl) amino-5-methylthieno [2, 3-d] pyrimidine derivatives as potent Mnk inhibitors: synthesis, structure–activity relationship analysis and biological evaluation. Eur. J. Med. Chem. 95, 116–126. 10.1016/j.ejmech.2015.03.032 25800647

[B92] Yu X.-H. Cai G.-J. Liu A.-J. Chu Z.-X. Su D.-F. (2007). A novel animal model for motion sickness and its first application in rodents. Physiol. Behav. 92, 702–707. 10.1016/j.physbeh.2007.05.067 17612582

