# Supplementary figures and images for: Brain Activation by H1 Antihistamines Challenges Conventional View of Their Mechanism of Action in Motion Sickness: A Behavioral, c-Fos and Physiological Study in Suncus murinus (House Musk Shrew)
Source: Front Physiol. 2017 Jun 14;8:412. doi: 10.3389/fphys.2017.00412 (PMC5470052; doi:10.3389/fphys.2017.00412)

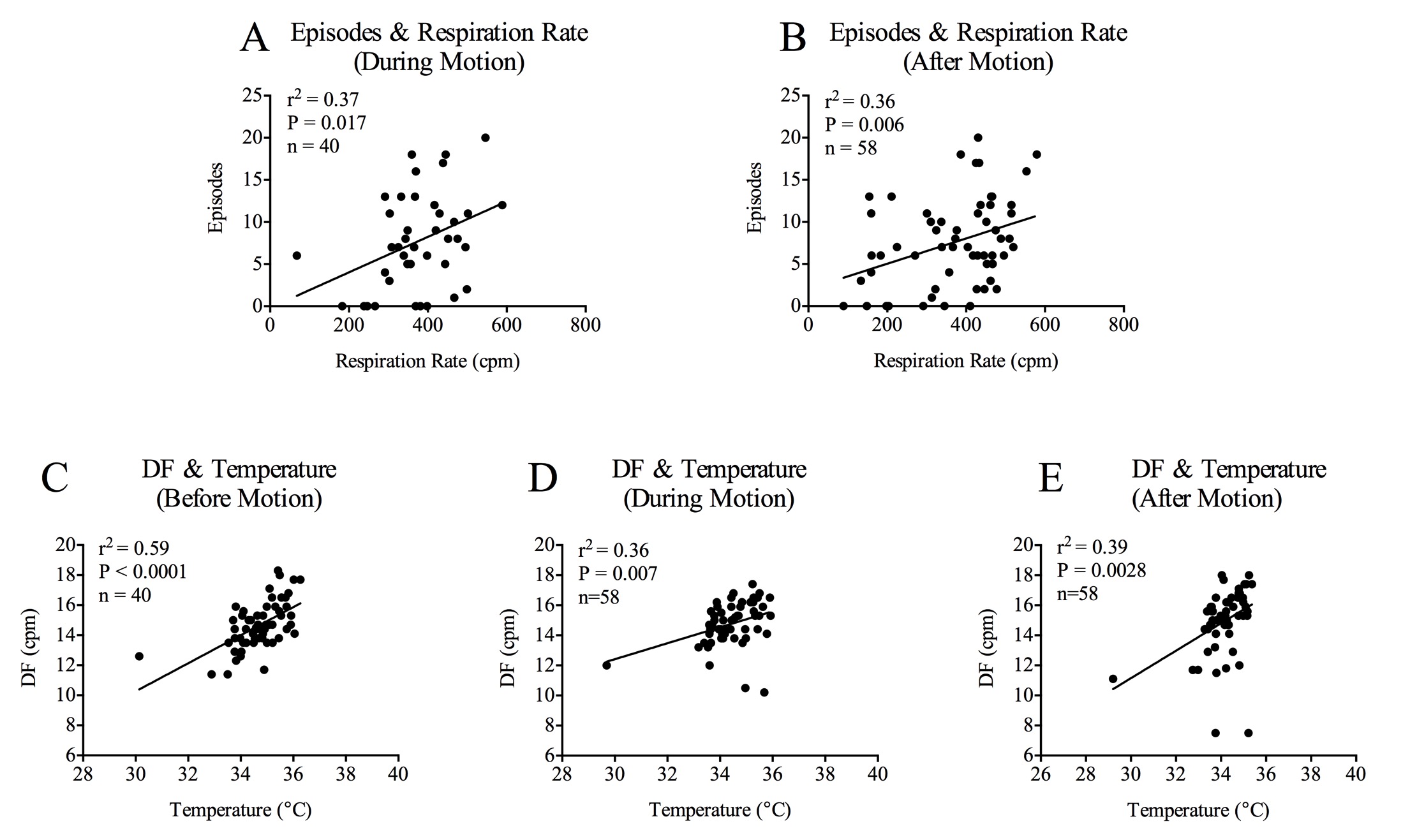

Supplement: Supplementary Figure 1 — Correlations of physiological parameters from pre-screening study. (A) Episodes vs. respiratory rate during motion; (B) Episodes vs. respiratory rate during recovery; (C) DF vs. core body temperature during baseline; (D) DF vs. core body temperature during motion; (E) DF vs. core body temperature during recovery. Data represents the mean ± s.e.m. of 40–52 animals. [file Image1.JPEG]

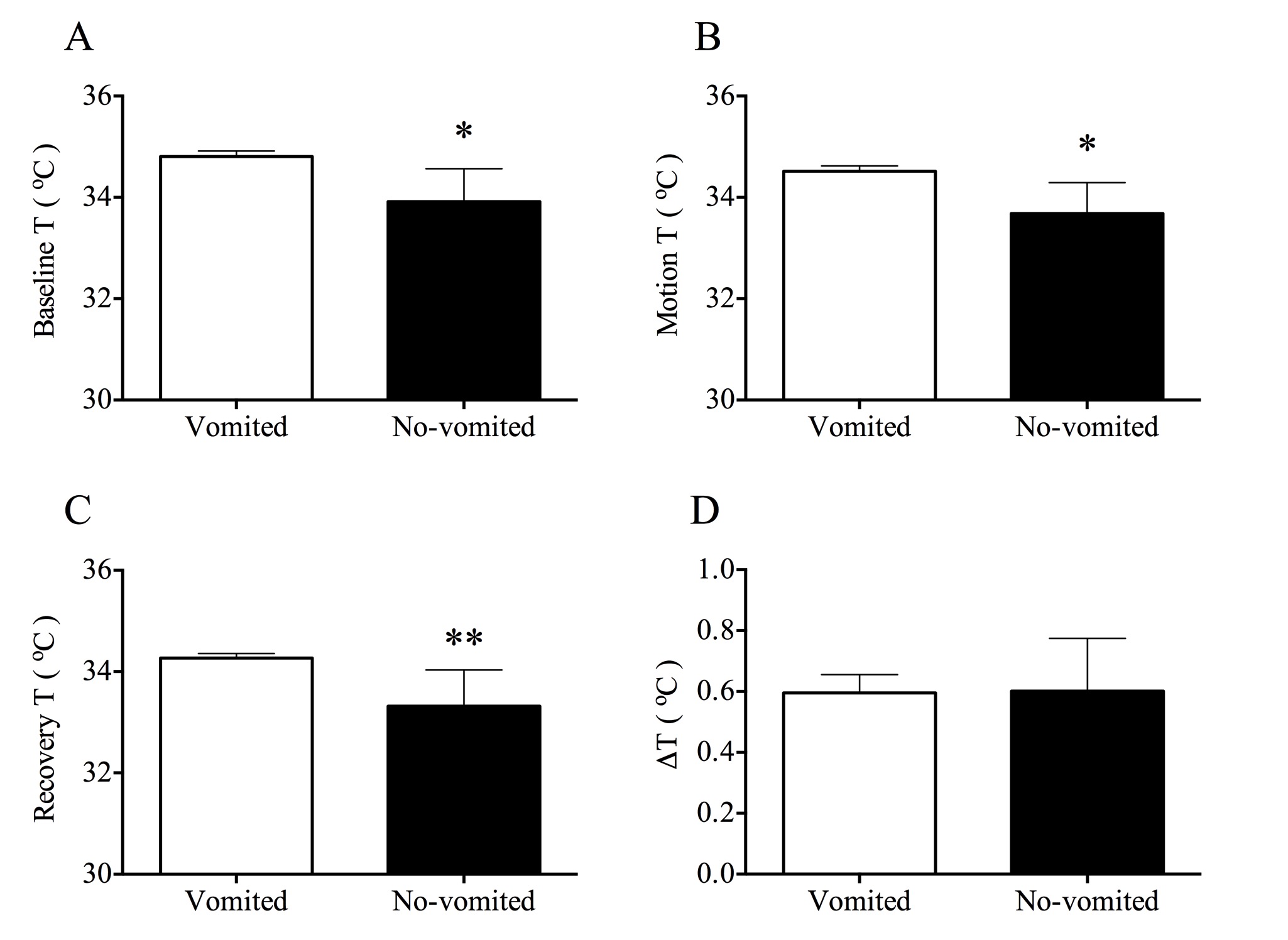

Supplement: Supplementary Figure 2 — Temperature difference between vomited and no-vomited animals during baseline, motion and recovery in pre-screening study. (A) Baseline; (B) Motion; (C) Recovery; (D) Δ temperature during motion. Data represents the mean ± s.e.m. of 51 vomited animals and 7 non-vomited animals. Significant differences are shown as *p < 0.05, **p < 0.01 (unpaired t-test). [file Image2.JPEG]

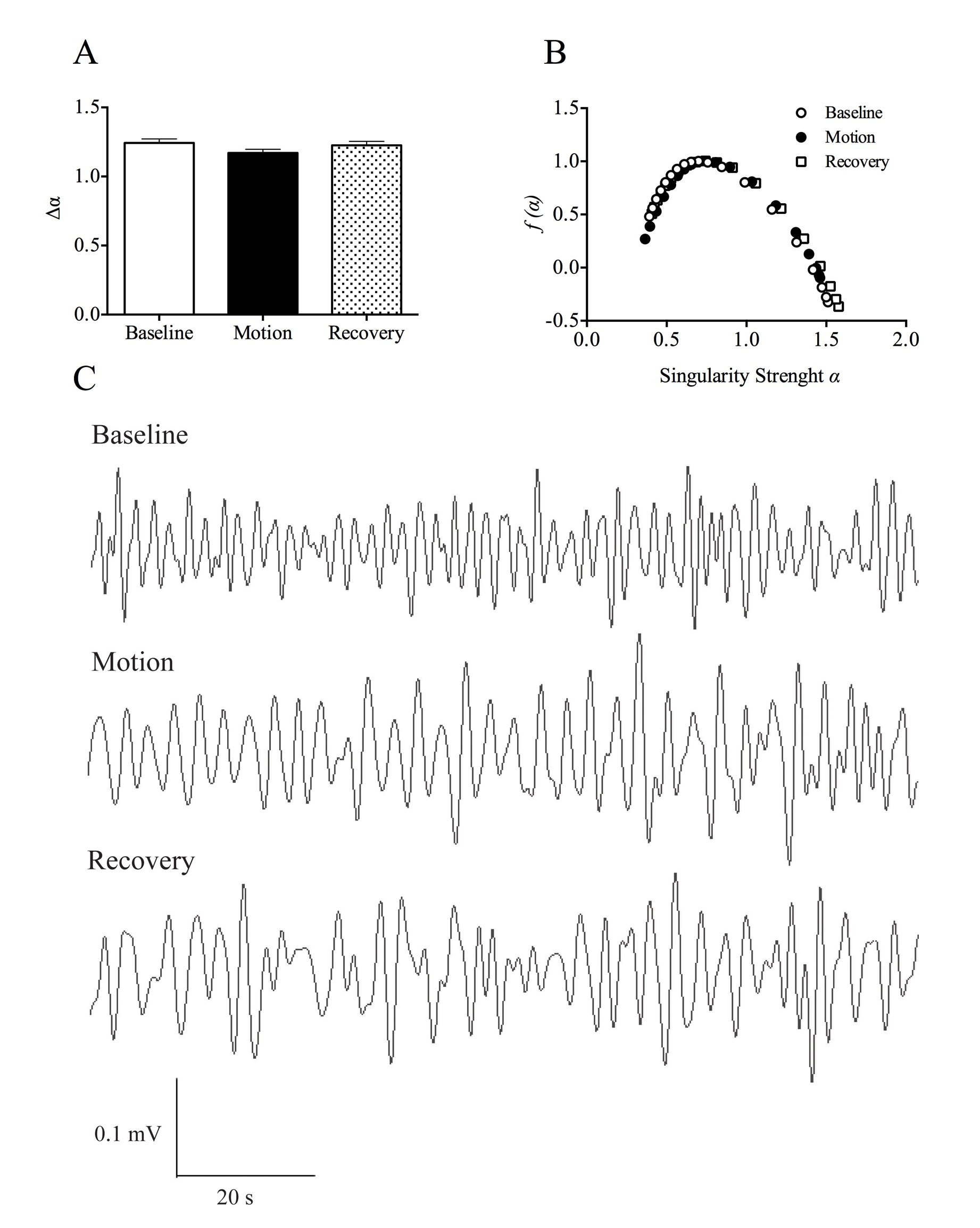

Supplement: Supplementary Figure 3 — Singularity spectra of time series of GMA that were estimated by multifractal detrended fluctuation analysis (MFDFA). (A) The width of singularity strength Δα of baseline, motion, and recovery during pre-screening study; (B) Graphs represents f (α) vs. singularity strength α; (C) representative raw traces of gastric slow waves from baseline, motion and recovery. Data represents the mean ± s.e.m. of 40–52 animals. [file Image3.JPEG]
