# Supplementary material for: Brain Activation by H1 Antihistamines Challenges Conventional View of Their Mechanism of Action in Motion Sickness: A Behavioral, c-Fos and Physiological Study in Suncus murinus (House Musk Shrew)
Source: Front Physiol. 2017 Jun 14;8:412. doi: 10.3389/fphys.2017.00412 (PMC5470052; doi:10.3389/fphys.2017.00412)
Supplement: Supplementary Table 1 — Effect of mepyramine (50 mg/kg), cetirizine (10 mg/kg), and scopolamine (10 mg/kg) on the width of singularity strength Δα of GMA. Data represents the mean ± s.e.m. of 6 animals. [file Table1.DOCX]

| Δα | Baseline | Motion | Recovery |
| --- | --- | --- | --- |
| Vehicle | 1.19 ± 0.12 | 1.16 ± 0.18 | 1.15 ± 0.05 |
| Mepyramine | 1.28 ± 0.06 | 1.25 ± 0.11 | 1.21 ± 0.03 |
| Cetirizine | 1.24 ± 0.10 | 1.16 ± 0.07 | 1.18 ± 0.08 |
| Scopolamine | 1.23 ± 0.15 | 1.32 ± 0.08 | 1.27 ± 0.08 |

**Supplementary Table 1.** Effect of mepyramine (50 mg/kg), cetirizine (10 mg/kg) and scopolamine (10 mg/kg) on the width of singularity strength ∆*α* of GMA.

Data represents the mean ± s.e.m. of 6 animals.
